# Predicting intelligence from brain gray matter volume

**DOI:** 10.1007/s00429-020-02113-7

**Published:** 2020-07-21

**Authors:** Kirsten Hilger, Nils R. Winter, Ramona Leenings, Jona Sassenhagen, Tim Hahn, Ulrike Basten, Christian J. Fiebach

**Affiliations:** 1 Department of Psychology, Goethe University Frankfurt, Frankfurt am Main, Germany; 2 Department of Psychology, Julius Maximilian University Würzburg, Würzburg, Germany; 3 IDeA Center for Individual Development and Adaptive Education, Frankfurt am Main, Germany; 4 Institute of Translational Psychiatry, University Hospital Münster, Münster, Germany; 5 Brain Imaging Center, Goethe University Frankfurt, Frankfurt am Main, Germany; 6 Present Address: Department of Psychology I, University Wuerzburg, Marcusstr. 9-11, 97070 Würzburg, Germany

**Keywords:** Intelligence, Gray matter volume, Voxel-based morphometry (VBM), Machine learning, Prediction, Brain size

## Abstract

A positive association between brain size and intelligence is firmly established, but whether region-specific anatomical differences contribute to general intelligence remains an open question. Results from voxel-based morphometry (VBM) - one of the most widely used morphometric methods - have remained inconclusive so far. Here, we applied cross-validated machine learning-based predictive modeling to test whether out-of-sample prediction of individual intelligence scores is possible on the basis of voxel-wise gray matter volume. Features were derived from structural magnetic resonance imaging data (*N* = 308) using (a) a purely data-driven method (principal component analysis) and (b) a domain knowledge-based approach (atlas parcellation). When using relative gray matter (corrected for total brain size), only the atlas-based approach provided significant prediction, while absolute gray matter (uncorrected) allowed for above-chance prediction with both approaches. Importantly, in all significant predictions, the absolute error was relatively high, i.e., greater than ten IQ points, and in the atlas-based models, the predicted IQ scores varied closely around the sample mean. This renders the practical value even of statistically significant prediction results questionable. Analyses based on the gray matter of functional brain networks yielded significant predictions for the fronto-parietal network and the cerebellum. However, the mean absolute errors were not reduced in contrast to the global models, suggesting that general intelligence may be related more to global than region-specific differences in gray matter volume. More generally, our study highlights the importance of predictive statistical analysis approaches for clarifying the neurobiological bases of intelligence and provides important suggestions for future research using predictive modeling.

**Electronic supplementary material:**

The online version of this article (10.1007/s00429-020-02113-7) contains supplementary material, which is available to authorized users.

## Introduction

Intelligence describes an individual’s ability to understand complex ideas, to adapt effectively to the environment, to learn from experience, and to engage in various forms of reasoning (Neisser et al. 1996). It is the best predictor of educational and occupational success (Neisser et al. 1996), relates closely to positive life outcomes like health and longevity (Deary et al. 2004), and is often defined as the general cognitive ability of a person. Understanding the neurobiological basis of intelligence is an important aim of ongoing research in the cognitive neurosciences.

By far the best-established neuroanatomical predictor of general intelligence is total brain size, accounting for up to 5% of variance in individuals’ intelligence quotients (Nave et al. 2018; Pietschnig et al. 2015). It has also been hypothesized that different brain regions may contribute differently to intelligence. For example, an influential model of the brain bases of intelligence, the parieto-frontal integration theory (P-FIT; Jung and Haier 2007) proposed that frontal and parietal cortices represent primary neural systems underlying inter-individual variation in general cognitive ability. Voxel-based morphometric methods (VBM; see, e.g., Ashburner and Friston 2000) have been used to examine the relationship between regionally specific differences in gray matter volume and intelligence at high spatial resolution (i.e., up to 1 mm), and early VBM studies (e.g., Haier et al. 2004) indeed support proposal role of parietal and frontal cortices for general intelligence. A recent coordinate-based quantitative meta-analysis of VBM studies from our research group, however, found only limited evidence for convergence of gray matter volume correlates of intelligence in parietal or frontal cortex across different studies (i.e., only very small clusters, no effects in lateral parietal cortex, and only when using rather lenient statistical thresholds; cf. Basten et al. 2015). The lack of consistent VBM findings may result from the widespread use of rather limited sample sizes (i.e., between 30 and 104 participants in studies included in the meta-analysis of Basten et al. 2015), and this situation is further complicated by the fact that not all VBM studies of regional gray matter correlates of intelligence differences controlled for the effect of individual differences in total brain size (see, e.g., Lee et al. 2005, as an example of a VBM study based on uncorrected gray matter volume data). Because total brain size is positively correlated with intelligence (Nave et al. 2018; Pietschnig et al. 2015), it is quite plausible to assume that also region-specific absolute gray matter volumes (approximating regional neuron numbers; Leuba and Kraftsik 1994) are associated with variations in intelligence. However, whether relative gray matter volumes, i.e., local deviations in gray matter volume beyond the global influence of total brain size, are correlated with intelligence is still an open question.

Additionally, all studies reviewed in our meta-analysis (as well as further studies not included in the meta-analysis due to, e.g., missing coordinates for effect localization) used an explanatory strategy in their statistical analysis approach. Such a strategy is prone to overfitting because statistical models are optimized to explain maximal amounts of variance within the respective samples but do not necessarily generalize to new out-of-sample data (see, e.g., Yarkoni and Westfall 2017, for an in-depth discussion). The introduction of predictive machine learning approaches to the field of neuroimaging (see, e.g., Lemm et al. 2011; Poldrack et al. 2020) has made it possible to explicitly test whether and to what extent neural features can predict a behavioral outcome measure (such as IQ), i.e., explain variance also in independent data. These predictive approaches - that include some form of cross-validation (i.e., an internal replication) - provide a less biased estimate of the generalization error, which reflects the extent to which associations are only valid in one specific sample but cannot be generalized to the population (Hastie et al. 2009; Yarkoni and Westfall 2017). Using such a predictive analysis approach, it has, for example, recently been demonstrated that individual differences in intelligence can be predicted from intrinsic (i.e., task independent) patterns of whole-brain functional connectivity based on resting-state fMRI, accounting for up to 25% of variation in behavioral measures of general cognitive ability (Dubois et al. 2018; Ferguson et al. 2017; Finn et al. 2015; Liu et al. 2018).

Here, we use predictive modeling to investigate whether individual intelligence scores can be predicted from regional differences in gray matter volume. To this end, we fit a cross-validated predictive model to voxel-based morphometric maps of gray matter volume using data from 308 adults whose Full-Scale Intelligence Quotient (FSIQ) was assessed with the Wechsler Abbreviated Scale of Intelligence (WASI; Wechsler 1999). On the one hand, this analysis was conducted after correcting for individual variations in total brain size (i.e., on relative regional gray matter volume data) to assess region-specific neuroanatomical correlates of intelligence beyond the known correlation between intelligence and total brain size. On the other hand, we also assessed whether intelligence can be predicted from regional gray matter volumes when not correcting for total brain size (i.e., from absolute gray matter volumes), to test the influence of total brain size on the prediction of intelligence from regional gray matter differences. As there exists no general consensus on how to best construct meaningful features from the very high-dimensional voxel-wise neuroimaging data, we implemented two different approaches of feature construction and compared the respective results: We started with a well-established and purely data-driven method, i.e., principal component analyses (PCA, see e.g., Abreu et al. 2019; Espinoza et al. 2019; Wasmuht et al. 2018). In addition, we implemented a more theoretically informed, domain knowledge-based approach, which combines voxel-specific gray matter values in regions of interest in accordance with a well-established functional brain atlas (Schaefer et al. 2018).

Beyond whole-brain prediction, it is also of interest to assess the predictive power of functionally defined brain networks for intelligence. This not only directly follows from neurocognitive models of intelligence like the parieto-frontal integration theory (Jung and Haier 2007) but is also motivated by more recent proposals highlighting the potential role that specific brain networks may play for general intelligence (Barbey 2018). Functional neuroimaging work has firmly established a set of functionally defined cortical networks (reviewed, e.g., in Dosenbach et al. 2006; Sporns and Betzel 2016; Yeo et al. 2011), and individual differences in intelligence have been associated with the fronto-parietal network (e.g., Barbey 2018; Hearne et al. 2016; Santarnecchi et al. 2017), the dorsal attention network centered on the intraparietal sulcus and the frontal eye fields (e.g., Hilger et al. 2020; Santarnecchi et al. 2017), the cingulo-opercular salience network (Barbey 2018; Hilger et al. 2017a, b; Santarnecchi et al. 2017), and the default mode network of the brain (Barbey 2018; Basten et al. 2013; Hearne et al. 2016; van den Heuvel et al. 2009). While recent correlative studies with large sample sizes indeed suggest associations with structural white matter connectivity (Genç et al. 2018) and with local gyrification (Gregory et al. 2016) in some of these systems, the role of network-specific individual differences in gray matter volume for intelligence has so far not been systematically explored. To fill this gap, we conducted all predictive analyses also independently for a set of well-defined functional brain networks.

## Methods

### Data and code availability

We used data from the Enhanced Rockland sample acquired by the Nathan S. Kline Institute for Psychiatric Research (NKI; Nooner et al. 2012), which was made available online as part of the 1000 Functional Connectomes Project via the International Neuroimaging Data-Sharing Initiative (INDI; https://fcon_1000.projects.nitrc.org/indi/enhanced/). The analysis code of our predictive modeling approach can be accessed online at https://github.com/NilsWinter/Predicting-Intelligence-From-Brain-Gray-Matter-Volume.

### Participants

All procedures were approved by the NKI Institutional Review Board (#239708) and informed written consent according to the Declaration of Helsinki was obtained from all participants. A subsample of 309 participants was selected for whom complete neuroimaging and phenotypical data were available, including the Wechsler Abbreviated Scale of Intelligence (WASI; Wechsler 1999). One participant was excluded on the basis of the CAT12 quality check due to problems in gray matter segmentation (see below), leaving a final sample of 308 participants (age 18–60 years, M = 38.87, SD = 13.92; 198 females; handedness assessed by the Edinburgh Handedness Questionnaire, EHQ, Oldfield 1971: 260 right, 22 left, 26 ambidextrous). The WASI Full-Scale Intelligence Quotient (FSIQ) ranged from 67 to 135 (M = 98.95, SD = 12.94).

### Structural magnetic resonance imaging and preprocessing

High-resolution structural images were acquired on a 3 T whole-body MRI scanner (MAGNETOM Trio Tim, Siemens, Erlangen, Germany) using a sagittal T1-weighted Magnetization Prepared-Rapid Gradient Echo (MP-RAGE) sequence with the following scanning parameters: 176 sagittal slices; voxel size 1 × 1 × 1 mm; TR 1900 ms; TE 2.5 ms; FOV 250 × 250 mm; flip angle 9°; acquisition time 4.18 min.

We generated individual maps of regional gray matter volume with the CAT12 toolbox (Computational Anatomy Toolbox version 10.73; https://www.neuro.uni-jena.de/cat/) for SPM12 (Statistic Parametric Mapping software, Welcome Department of Imaging Neuroscience, London, UK). T1-weighted images were segmented into gray matter, white matter, and cerebrospinal fluid. Dartel (Diffeomorphic Anatomical Registration Through Exponentiated Lie Algebra; Ashburner 2007) was used for spatial normalization to the MNI152 (Montreal Neurological Institute) template and to determine the parameters of the nonlinear deformations. These parameters were then used to correct the normalized gray matter probability maps for local volume changes induced by the normalization step and to generate *m*-modulated gray matter probability maps (corrected for non-linear and linear/affine components by multiplication with the Jacobian determinant; Good et al. 2001). Then, a quality check was performed to ensure sample homogeneity of gray matter tissue (see CAT12 manual; Gaser and Kurth 2018). This led to the exclusion of one subject.

**Fig. 1:**
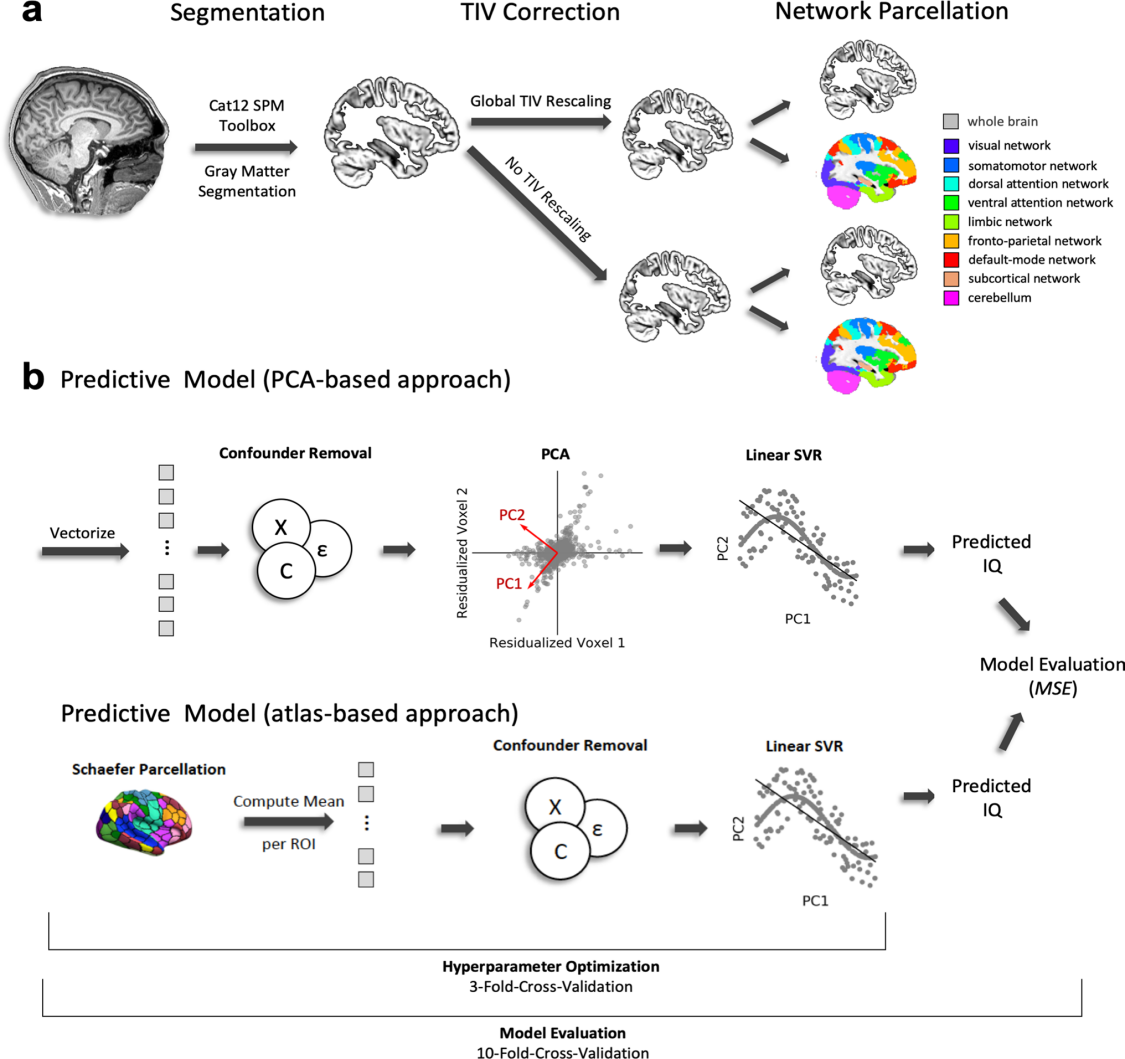
Schematic illustration of processing steps and analysis workflow. **a** Preprocessing of structural MRI data. T1-weighted MR images were segmented using the CAT12 SPM toolbox to generate individual gray matter volume maps, which were then corrected and rescaled for between-person variations in total intracranial volume (TIV, see “Methods” for further details). The TIV-rescaled gray matter volume maps (representing relative gray matter volume) and the raw gray matter volume maps (uncorrected for TIV, representing absolute gray matter volume) were used to establish a global (whole-brain) prediction model. In addition, all maps were parcellated into the seven established functional brain networks (derived from Yeo et al. 2011). A subcortical network and the cerebellum were added in the PCA-based approach only. Together with the whole-brain model, this resulted in ten analyses for the TIV-rescaled (relative) and ten analyses for the non-rescaled (absolute) regional gray matter volume data for the PCA-based approach. In respect to the atlas-based approach, this resulted in eight analyses for relative and eight analyses for absolute gray matter data. **b** In these analyses, the vectorized data were fed to our predictive model. In the PCA-based approach, the first step consisted of removing confounder variables (age, sex, handedness) from every voxel using linear regression. Then a full variance decomposition (PCA) was performed on the residualized data. The resulting principal components were then used as input for a linear SVR to predict the IQ score of individual subjects. In the atlas-based approach, the data were at first parcellated into 400 parcels in accordance with the Schaefer atlas (Schaefer et al. 2018), then gray matter values were averaged within each parcel, and lastly, residualized in respect to the control variables. The resulting averaged gray matter volume values were fed into a linear SVR and predicted IQ scores were computed. Importantly, the hyperparameters of the SVR were optimized using a threefold cross-validation which was nested inside a tenfold cross-validation scheme to evaluate the final model performance with mean squared error as a primary model evaluation criterion (see also Supplementary Figure S1 for a more detailed visualization). *TIV* total intracranial volume, *PCA* principal component analysis, *PC* principal component, *SVR* support vector regression, *IQ* intelligence quotient, *MSE* mean squared error

To examine regionally specific effects of gray matter volume independent of total brain size (i.e., relative gray matter volume), the *m*-modulated gray matter probability maps were corrected for total intracranial volume (TIV) by global rescaling (Fig. 1a). Rescaling is recommended when TIV significantly correlates with the variable of interest, i.e., the target of the prediction model, in this case, intelligence (Gaser and Kurth 2018). The existence of an association between TIV and intelligence is an established finding (see above; McDaniel 2005; Nave et al. 2018; Pietschnig et al. 2015), and also present in the current dataset; we observed significant associations between FSIQ and TIV (*r* = 0.22, *p* < 0.001), between FSIQ and mean absolute gray matter volume (i.e., averaged across all voxels; *r* = 0.18, *p* = 0.002), and between mean absolute gray matter volume and TIV (*r* = 0.82, *p* < 0.001). We thus rescaled the gray matter value of each voxel by (1) dividing it by the subject’s individual TIV value and then (2) multiplying the result with the mean TIV value of the whole group. This resulted in one image of relative regional gray matter volume per subject, each of which consisted of 556,694 voxels, which served as input for the multivariate analyses. Note that after TIV rescaling, the correlation between FSIQ and relative mean gray matter volume was not significant anymore (*r* = 0.07, *p* = 0.229). With the aim of comparing the predictive performance between relative (i.e., TIV-rescaled) and absolute gray matter volumes, we conducted the same analysis also without rescaling.

### Multivariate analyses

Predictive analyses were conducted using PHOTON, a python-based hyperparameter optimization and evaluation framework for rapid prototyping in machine learning (Leenings et al. 2020). We implemented a machine learning pipeline comprising two different methods of feature construction (PCA-based vs. atlas-based), confound regression, and a final multivariate estimator (involving hyperparameter optimization and a nested cross-validation scheme). Schematic illustrations of the multivariate analysis workflow are presented in Fig. 1b and, for a more detailed illustration of the nested cross-validation scheme, in Supplementary Fig. S1.

#### Feature construction

As outlined above, we implemented two different methods of feature construction, i.e., PCA vs. atlas-based. Both of these methods comprise two steps of feature transformation. First, although age, sex, and handedness were not significantly correlated with intelligence in our sample (age: *r* = 0.05, *p* = 0.42; sex: *r* = − 0.06, *p* = 0.30; handedness: *r* = − 0.01, *p* = 0.80), we decided to control for these variables in both approaches to achieve comparability with former VBM research reporting associations between intelligence and gray matter volumes (e.g., Colom et al. 2013; Haier et al. 2004) and with recent investigations predicting intelligence from brain function (e.g., Dubois et al. 2018). Thus, we residualized the individual gray matter volume values with respect to these variables within our cross-validated machine learning pipeline using linear regression as implemented in Python’s statsmodels package. For the PCA-based approach, this was done before reducing the dimensionality of the data with PCA. PCA is a statistical procedure that transforms the data into a restricted number of orthogonal components capturing the most essential parts of variance in the original data. As the number of features in the data (i.e., one gray matter volume value per voxel) is in our case always larger than the number of subjects in the training set, the latter determined the maximum number of extracted principal components (i.e., 277 or 278). In the second approach, we used the Schaefer parcellation (Schaefer et al. 2018) and first reduced the dimensionality of our feature space by averaging gray matter volume values across voxels within the 400 parcels provided by this atlas. These averaged gray matter volume values were then residualized with respect to the control variables specified above. The resulting features (principal components vs. averaged gray matter values) were then used as input to a Support Vector Regression (SVR) as implemented in Scikit Learn (https://scikit-learn.org/stable/modules/svm.html; Boser et al. 1992; Drucker et al. 1997; Noble 2007). SVR is an extension of Support Vector Classification to continuous data and relies on a regularization process with an *ε*-insensitive (hinge-loss) cost function. For all analyses, individual FSIQ scores served as targets.

#### Hyperparameter optimization

The optimization of hyperparameters is of crucial importance when building a machine learning pipeline and numerous strategies have evolved to efficiently find the optimal solution (Bergstra and Bengio 2012; Snoek et al. 2012). We used a Bayesian optimization strategy as implemented in the Scikit Optimize library (https://zenodo.org/record/1207017#.XTA0EpMzZp8; Head et al. 2018) which is also available in PHOTON (Leenings et al. 2020). Within Scikit Optimize, a Gaussian Process Regression was used as the base estimator to identify the configuration of SVR hyperparameters that minimizes the mean squared error (MSE) of the overall predictive model. We ran 50 evaluations of which, by default, ten were used as initialization points before approximating the hyperparameter space with the base estimator. The SVR hyperparameters we optimized were the regularization terms *ε* and *C* that define the trade-off between penalizing the model for points outside a tube of equivalence (zero-loss) around the hyperplane (the width of that tube is defined by *ε*) vs. penalizing the model for the distance of each point from the hyperplane (Smola and Schölkopf 2004). Regularization rewards parsimonious models (which usually generalize better to unseen data) vs. more complex models that capture the training data well but often do not generalize to unseen data (overfitting). We set the possible range of the *ε* parameter from 0.01 to 3 (default value of Scikit Learn: 0.1, larger values depict a larger zero-loss *ε*-tube) and allowed the *C* parameter to vary between 1e−6 and 1 (default value of Scikit Learn: 1, smaller values increase the regularization). For all other parameters of the SVR, the default settings of Scikit Learn were used.

#### Cross-validation

We used a strictly nested cross-validation scheme as implemented in PHOTON, with stratified folds to ensure a homogeneous distribution of intelligence scores across all folds. An outer loop (tenfold, *N*_train_ = 277 or 278, *N*_test_ = 31 or 30) was implemented to determine the model fit, while an inner loop was used to optimize - within each of the outer loop’s ten training folds - the hyperparameters of the pipeline (threefold, *N*_train_ = 184/185 and *N*_test_ = 93/92 for *N*_train_ = 277 in the outer loop, *N*_train_ = 185/186 and *N*_test_ = 93/92 for *N*_train_ = 278 in the outer loop; see Fig. S1 for schematic illustration). Importantly, this nested cross-validation approach avoids any information leakage from data of the training set into data of the test set. In other words, optimizing the hyperparameters within a nested cross-validation scheme ensures that every transformation step of the hyperparameters is performed exclusively on the training sample and that only the final set of hyperparameters is subsequently applied to the test set. This process allowed us to obtain an unbiased estimate of model performance (and the generalization error).

#### Specification of global vs. local prediction models

**Fig. 2:**
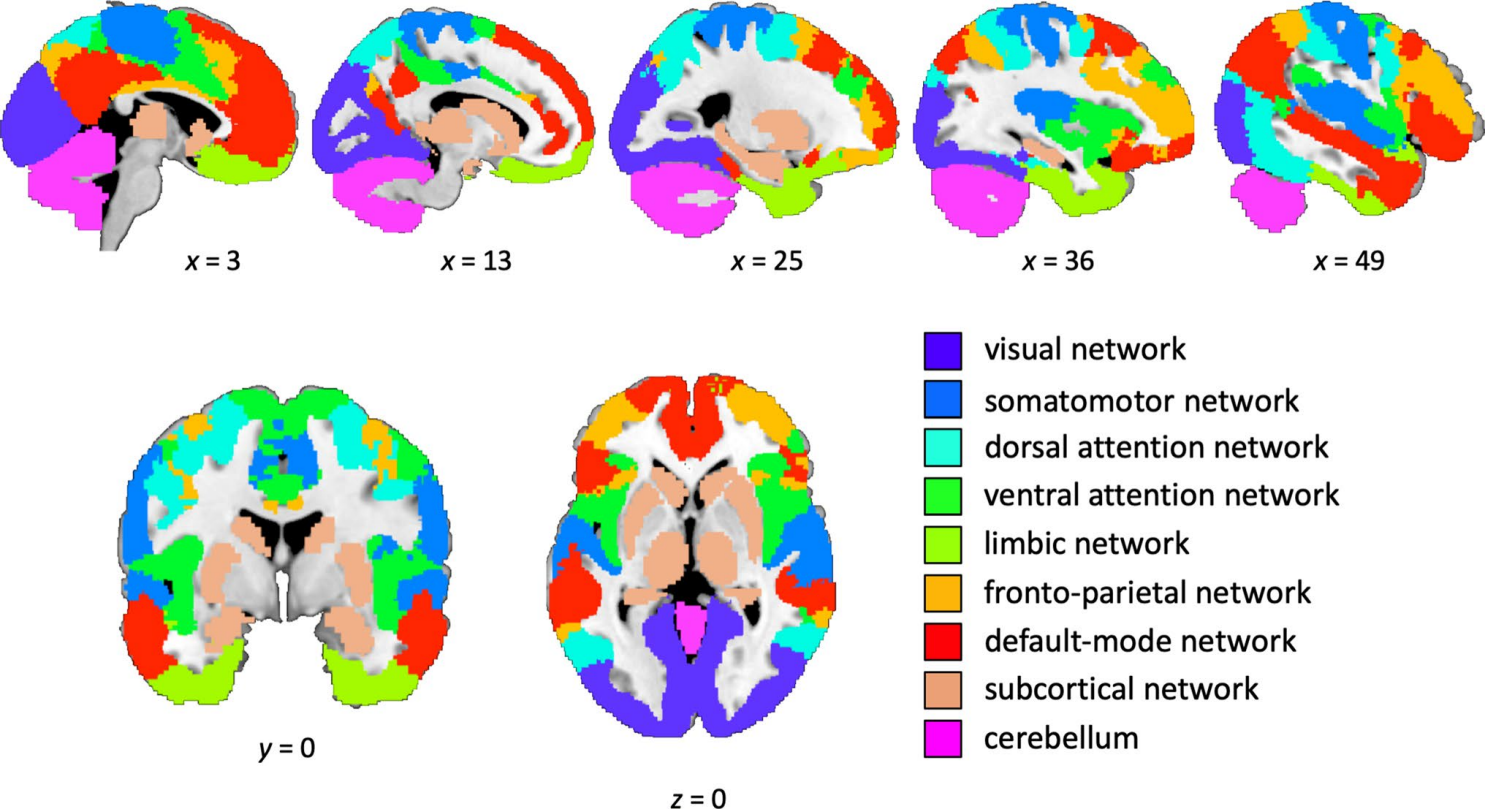
Anatomical location of functional brain networks. The figure illustrates the anatomical location of the nine functional networks that were used for the local models. Seven networks were derived from the Yeo atlas (Yeo et al. 2011; 7-network parcellation, liberal mask). In the PCA-based approach, a mask for the cerebellum and a subcortical module comprising putamen, caudate nucleus, thalamus, hippocampus, and amygdala (both derived from the Automatic Anatomical Labeling atlas, AAL; Tzourio-Mazoyer et al. 2002) were added. The *x*-, *y*- and *z*-coordinates represent coordinates of the Montreal Neurological Institute template brain (MNI152)

As outlined in Sect. 1, we implemented two different approaches to test whether intelligence can be predicted from patterns of gray matter volume. First, we tested whether the prediction of intelligence from gray matter volume was generally possible using data of all 556,694 voxels in the whole brain to construct the (PCA-based or atlas-based) model features. Second, we then also investigated whether the prediction of intelligence was driven by specific (i.e., functionally separable) brain networks (or brain modules; e.g., Sporns and Betzel 2016). To this end, we parcellated each normalized individual brain into distinct functional networks (Fig. 2, see also Fig. 1a). These networks were derived from the Yeo atlas describing seven networks of intrinsically coupled brain regions, for which a functional interpretation is available (Yeo et al. 2011; 7-network parcellation, liberal mask). For the PCA-based approach, we added the cerebellum and a subcortical network comprising putamen, caudate nucleus, thalamus, hippocampus, and amygdala, because both subcortical and cerebellar brain regions have previously been suggested as being relevant for intelligence (e.g., Basten et al. 2015; Burgaleta et al. 2014; Saxe et al. 2018). Masks for the subcortical network and the cerebellum were derived from the Automatic Anatomical Labeling atlas (AAL, Tzourio-Mazoyer et al. 2002) as implemented in the WFU PickAtlas (https://fmri.wfubmc.edu/software/pickatlas; Maldjian et al. 2003). For the atlas-based approach, it was not possible to include the cerebellum and subcortical network, as the Schaefer 400 parcels cover cortical regions only. Accordingly, also the global (whole-brain) atlas-based model did not encompass these regions. For these local, network-specific analyses, the analysis pipeline described above was applied separately to the data of each of these networks. This resulted in nine local predictive models for the PCA-based approach and in seven local models for the atlas-informed approach. As already specified above, we conducted the whole-brain and the network-specific analyses once for relative gray matter values, and once for absolute gray matter values (see also below), and by using both feature construction approaches, i.e., PCA vs. atlas-based.

#### Model evaluation

A tenfold cross-validation in the outer fold was used in both approaches to assess the model fit. The MSE served as the global index of model quality as this measure captures differences in bias and precision. To evaluate the predictive models, MSE values were averaged across all folds. For interpretation purposes, we also calculated the mean absolute error (MAE) and the root mean squared error (RMSE), both averaged across folds, which provide direct information about the expected average error in IQ points that we would make when predicting IQ scores of individual persons. For comparability with previous studies, we also computed the Pearson’s correlation coefficient (*r*) between predicted and observed FSIQ values (reported in Table 1). As each of the cross-validation folds predicted FSIQ scores for 31 (i.e., in eight folds) or 30 subjects (i.e., in two folds), the correlation coefficients were computed separately for each fold and subsequently averaged across all folds (after Fisher’s *z* transformation). The MSE was also used as the evaluation metric in the hyperparameter optimization (inner fold).

**Table 1:** Results of prediction models based on relative gray matter volume for the PCA-based approach (first row) and for the atlas-informed feature construction method (second row)

	Network size	MSE	*p*_perm_	Range	MAE	RMSE	*r*
Global model	556,694 400	320 197	0.279 < 0.001*	156–987 158–232	13.98 11.35	17.13 14.05	0.11 0.11
Local models							
Visual network	52,753 61	182 213	0.010 0.260	120–264 175–241	10.92 11.69	13.40 14.57	− 0.18 0.06
Somatomotor network	46,282 77	204 210	0.168 0.102	149–251 168–235	11.14 11.59	14.23 14.49	0.07 0.11
Dorsal attention network	36,374 46	193 208	0.030 0.008	122–242 170–224	11.14 11.56	13.84 14.41	0.17 0.19
Ventral attention network	32,345 47	202 212	0.162 0.199	141–265 174–240	11.15 11.66	14.17 14.56	0.06 − 0.02
Limbic network	27,296 26	219 212	0.438 0.160	181–260 175–234	12.07 11.63	14.76 14.56	0.03 0.16
Fronto-parietal network	45,921 52	191 205	0.035 < 0.001*	125–232 172–230	11.33 11.49	13.76 14.33	0.13 0.18
Default-mode network	71,492 91	181 208	0.008 0.017	127–240 171–230	11.00 11.56	13.38 14.41	0.22 0.22
Subcortical network	20,361 –	184 –	0.006 –	80–254 –	11.03 –	13.36 –	0.21 –
Cerebellum	57,851 –	171 –	< 0.001* –	146–195 –	10.42 –	13.07 –	0.27 –

Network size is depicted in number of voxels for the PCA-based approach and in number of parcels for the atlas-based feature construction method. Note, that in the PCA-based approach the number of features was independent of network size, i.e., features were always 277/278 principal components, whereas in the atlas-based approach the number of features corresponds to the number of parcels, i.e., the network size. Results indicating statistical significance are marked with an asterisk (Bonferroni-corrected for multiple comparisons). *MSE* mean squared error, *p*_*perm*_* p *value of statistical significance computed by non-parametric permutation test, *range* of MSE values resulting from different cross-validation folds, *MAE* mean absolute error in IQ points, *RMSE* root mean squared error in IQ points, *r* Pearson’s correlation coefficient between predicted and observed Full-Scale Intelligence Quotient (FSIQ) scores. All model fit indices were calculated for each cross-validation fold separately and averaged across folds afterwards

Because it has been shown that parametric statistical tests could lead to biased estimates of significance and false-positive or false-negative results in cross-validated prediction models (Combrisson and Jerbi 2015; Noirhomme et al. 2014), statistical significance of above-chance predictive performance was assessed with a non-parametric permutation test for all models. More specifically, we took the 308 targets (FSIQ scores) and permuted those values, which resulted in a random assignment of persons to FSIQ scores. Next, predictive performance (MSE) was assessed for these permuted targets. This step was repeated 1000 times. Finally, we summed the number of times for which model performance based on the true targets was lower than the performance for the permuted targets. *p* values for each model were derived by dividing this number by the number of permutations, i.e., 1000. Statistical significance was indicated by *p* values < 0.05 for the global model, by *p* values < 0.0056 for the nine local models of the PCA-based approach (nine comparisons, Bonferroni-corrected for multiple comparisons), and by *p* values < 0.0071 for the seven local models of the atlas-informed approach (seven comparisons, Bonferroni-corrected for multiple comparisons).

## Results

### Predicting intelligence from whole-brain relative gray matter volume

**Fig. 3:**
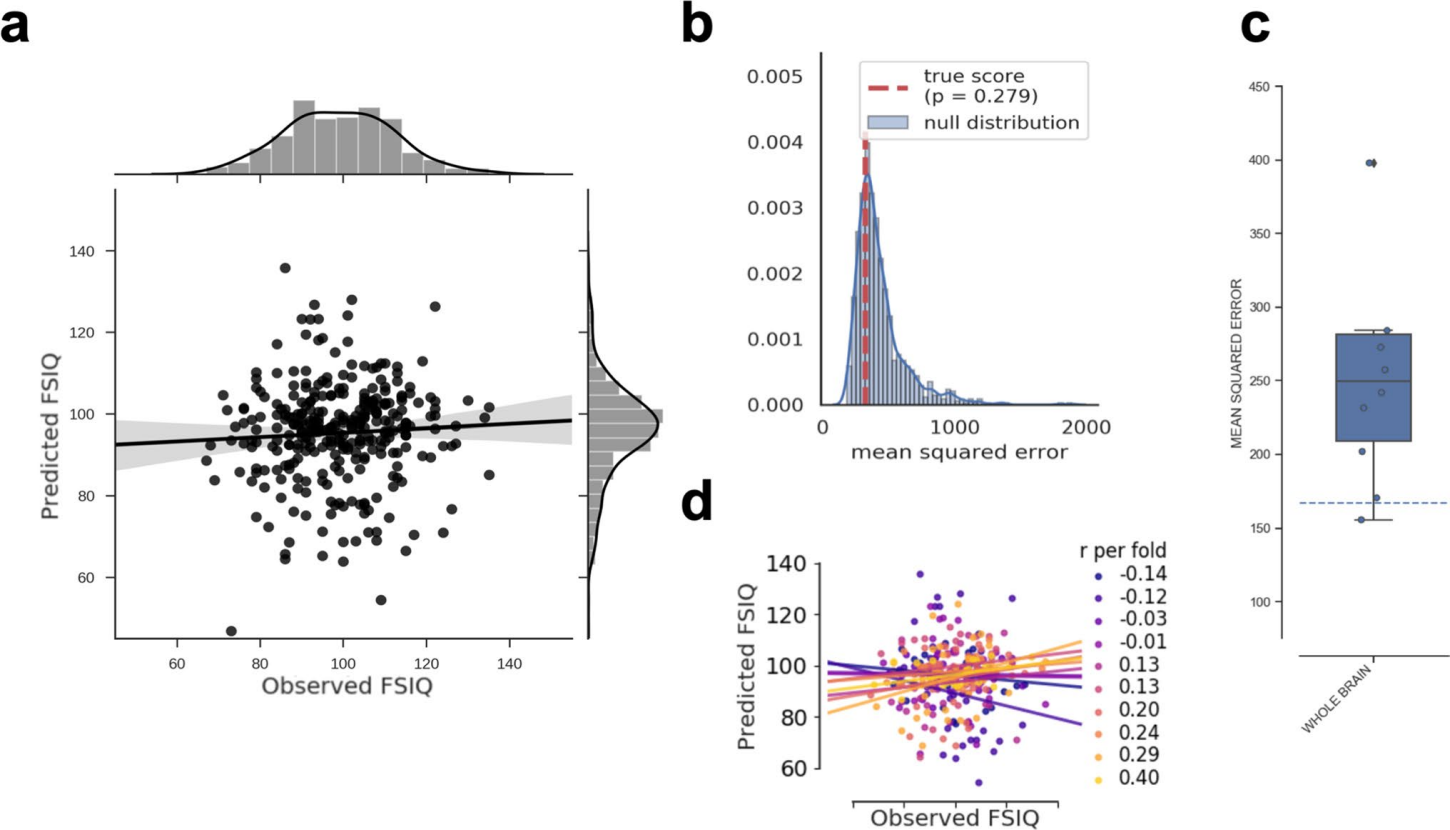
Predictive performance of the global model based on relative (i.e., TIV-rescaled) gray matter volume and the PCA-based feature construction approach. **a** Observed (*x*-axis) vs. predicted (*y*-axis) Full-Scale Intelligence Quotient (FSIQ) scores for all 308 participants. The gray area around the regression line represents the 95%-confidence interval (determined by bootstrapping) of prediction accuracy. Note that to allow the same scaling of *y*-axes as in the local models (Fig. 5), one data point was removed only for illustration. **b** Results of the non-parametric permutation test. The histogram shows the predictive performance given surrogate-null data, i.e., the distribution of the test statistic (mean squared error, MSE) based on permuted data (*N* = 1000 permutations; blue line: KDE smoothing) in relation to the predictive performance (MSE) based on the observed (non-permuted) data (red vertical line). If the MSE of the observed data had occurred in the extreme tails of the surrogate/permuted data, the prediction result from the machine learning pipeline would have been highly unlikely to be generated by chance, and thus considered significant. The *p* value resulted from summing up the times in which model performance based on the true targets was lower than model performance based on the permuted targets and dividing this number by the number of permutations. Thus, *p* values correspond to the percentile position of the observed MSE in the distribution of surrogate-null values. **c** Boxplot illustrating the variability of predictive performance (MSE) across folds. The boxes represent the interquartile range, horizontal lines represent the median, and the whiskers extend to points that lie within 1.5 times the interquartile ranges. The dotted line illustrates the performance of a ‘dummy model’ predicting the group-mean IQ of the training sample for every subject of the test sample. Note that for illustration only one data point was deleted (at MSE = 1000) to enable the same scaling of the *y*-axis for all boxplots in the paper. **d** Fold-wise illustration of the correlation between observed versus predicted FSIQ scores for all 308 participants. Predictions of each cross-validation fold and the corresponding approximated linear regression slopes are highlighted in different colors. *FSIQ* Full-Scale Intelligence Quotient, *r* Pearson’s correlation coefficient between predicted and observed FSIQ score

**Fig. 4:**
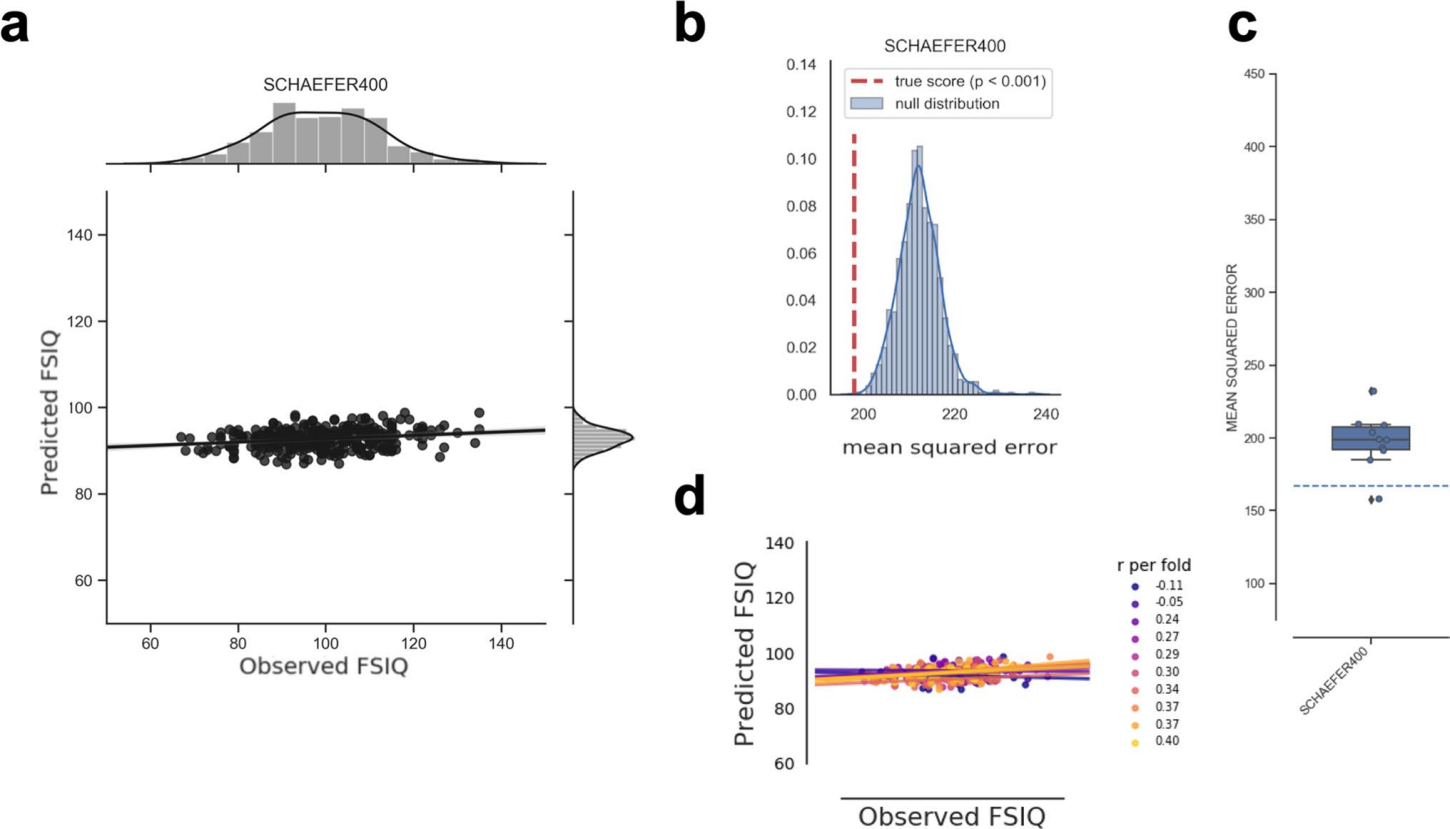
Predictive performance of the global model based on relative (i.e., TIV-rescaled) gray matter volume and the atlas-based feature construction approach. **a** Observed (*x*-axis) versus predicted (*y*-axis) Full-Scale Intelligence Quotient (FSIQ) scores for all 308 participants. The gray area around the regression line represents the 95% confidence interval (determined by bootstrapping) of prediction accuracy. Note that to allow the same scaling of *y*-axes as in the local models (Fig. 6), one data point was removed only for illustration. **b** Results of the non-parametric permutation test. The histogram shows the predictive performance given surrogate-null data, i.e., the distribution of the test statistic (mean squared error, MSE) based on permuted data (*N* = 1,000 permutations; blue line: KDE smoothing) in relation to the predictive performance (MSE) based on the observed (non-permuted) data (red vertical line). If the MSE of the observed data had occurred in the extreme tails of the surrogate/permuted data, the prediction result from the machine learning pipeline would have been highly unlikely to be generated by chance, and thus considered significant. The *p* value resulted from summing up the times in which model performance based on the true targets was lower than model performance based on the permuted targets and dividing this number by the number of permutations. Thus, *p* values correspond to the percentile position of the observed MSE in the distribution of surrogate-null values. **c** Boxplot illustrating the variability of predictive performance (MSE) across folds. The boxes represent the interquartile range, horizontal lines represent the median, and the whiskers extend to points that lie within 1.5 times the interquartile ranges. The dotted line illustrates the performance of a ‘dummy model’ predicting the group-mean IQ of the training sample for every subject of the test sample. **d** Fold-wise illustration of the correlation between observed versus predicted FSIQ scores for all 308 participants. Predictions of each cross-validation fold and the corresponding approximated linear regression slopes are highlighted in different colors. *FSIQ* Full-Scale Intelligence Quotient, *r* Pearson’s correlation coefficient between predicted and observed FSIQ score

We first investigated whether intelligence can be predicted from multivariate patterns of relative regional gray matter volume with a global model taking into account gray matter volume values of all voxels in the entire brain. PCA was used to reduce the number of features separately within each cross-validation fold. This model could not predict intelligence, i.e., predictive performance of the model was not significantly better than chance (MSE = 320, *p* = 0.279, see Table 1 and Fig. 3a; for results of the non-parametric permutation test, see Fig. 3b; for fold-wise predictive performance, see Fig. 3c, d). Similar results were obtained when assessing model fit with MAE (13.98, see Table 1, Fig. S2A) or RMSE (17.13, see Table 1, Fig. S2C), and the Pearson’s correlation coefficient between predicted and observed IQ scores was *r* = 0.11 (range of predicted scores: 39–136 IQ points). In contrast, the whole-brain model built on averaged gray matter values within the 400 parcels from the Schaefer atlas (Schaefer et al. 2018; atlas-based approach) achieved significant prediction of intelligence (MSE = 197, *p* < 0.001, see Table 1; for scatterplot of predicted vs. observed IQ scores, see Fig. 4a; for results of the non-parametric permutation test, see Fig. 4b; for fold-wise predictive performance, see Fig. 4c, d). However, Fig. 4a shows that the predicted FSIQ values are distributed very narrowly around the sample mean (range of predicted scores: 87–99 IQ points), which calls into question the practical relevance of the prediction result despite achieving statistical significance. This is further supported by the fact that the mean absolute error (MAE = 11.35, Table 1, Fig. S3A) and root mean squared error (RMSE = 14.05, Table 1, Fig. S3C) were only slightly improved compared to the PCA-based analysis approach, and a similar correlation coefficient was obtained (*r* = 0.11). The restricted range of predicted IQ scores also resulted in greatly reduced variance between prediction folds (Fig. 4c, d).

### Predicting intelligence from network-specific relative gray matter volume

**Fig. 5:**
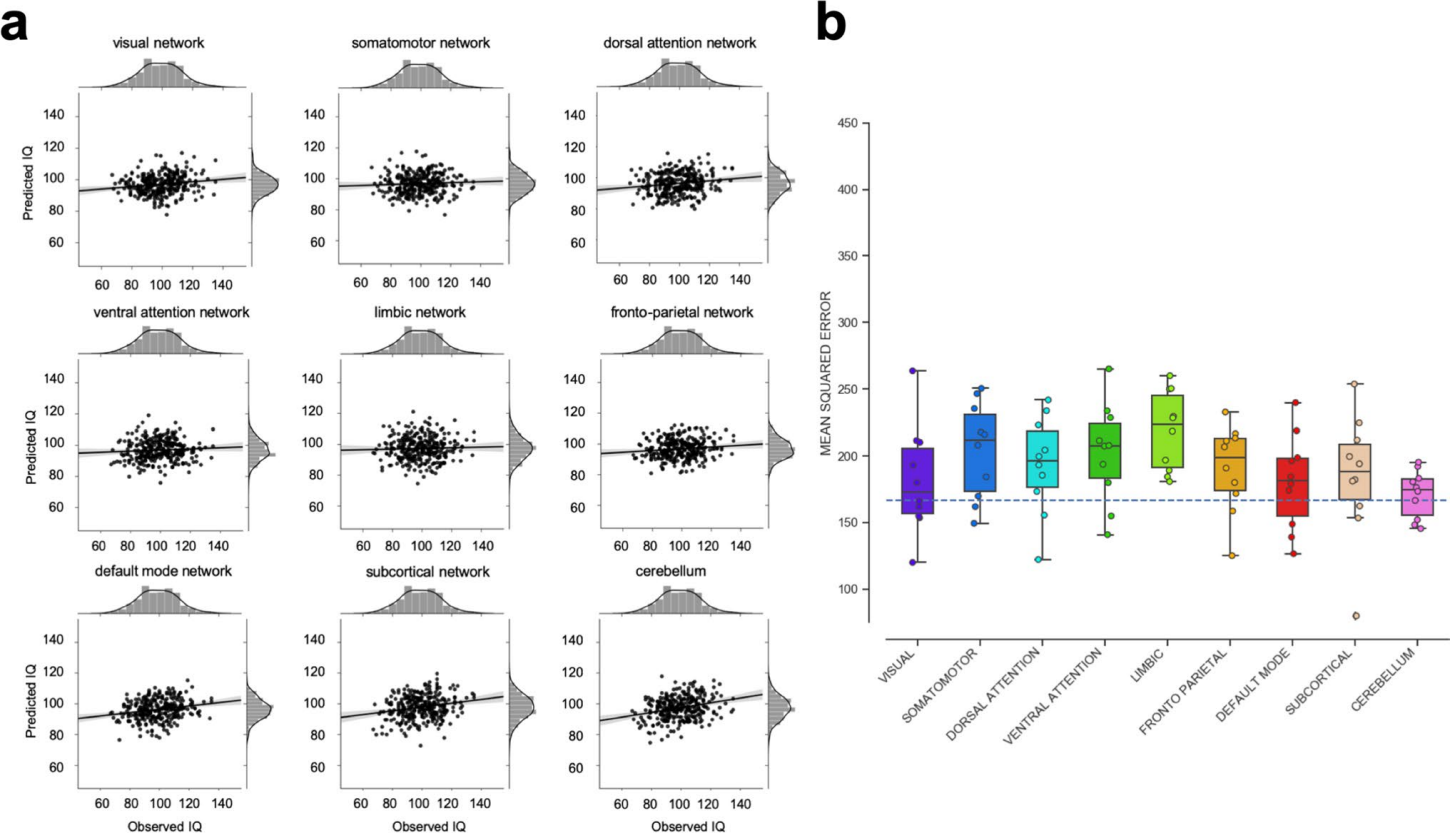
Predictive performance of nine local models based on relative (i.e., TIV-rescaled) gray matter volume and the PCA-based feature construction approach. The nine local models represent the functionally defined brain networks depicted in Fig. 2 (see also “Methods” for further details). **a** Observed (*x-*axis) versus predicted (*y*-axis) FSIQ scores for all 308 participants, separately for each functional network. The gray areas around the regression lines represent the 95%-confidence intervals (determined by bootstrapping) of prediction accuracies. **b** Boxplots illustrating the variability of predictive performance (mean squared error, MSE) across cross-validation folds. The boxes represent the interquartile range, horizontal lines represent the median, and the whiskers extend to points that lie within 1.5 times the interquartile ranges. The dotted line illustrates the performance of a ‘dummy model’ predicting the group-mean IQ of the training sample for every subject of the test sample

**Fig. 6:**
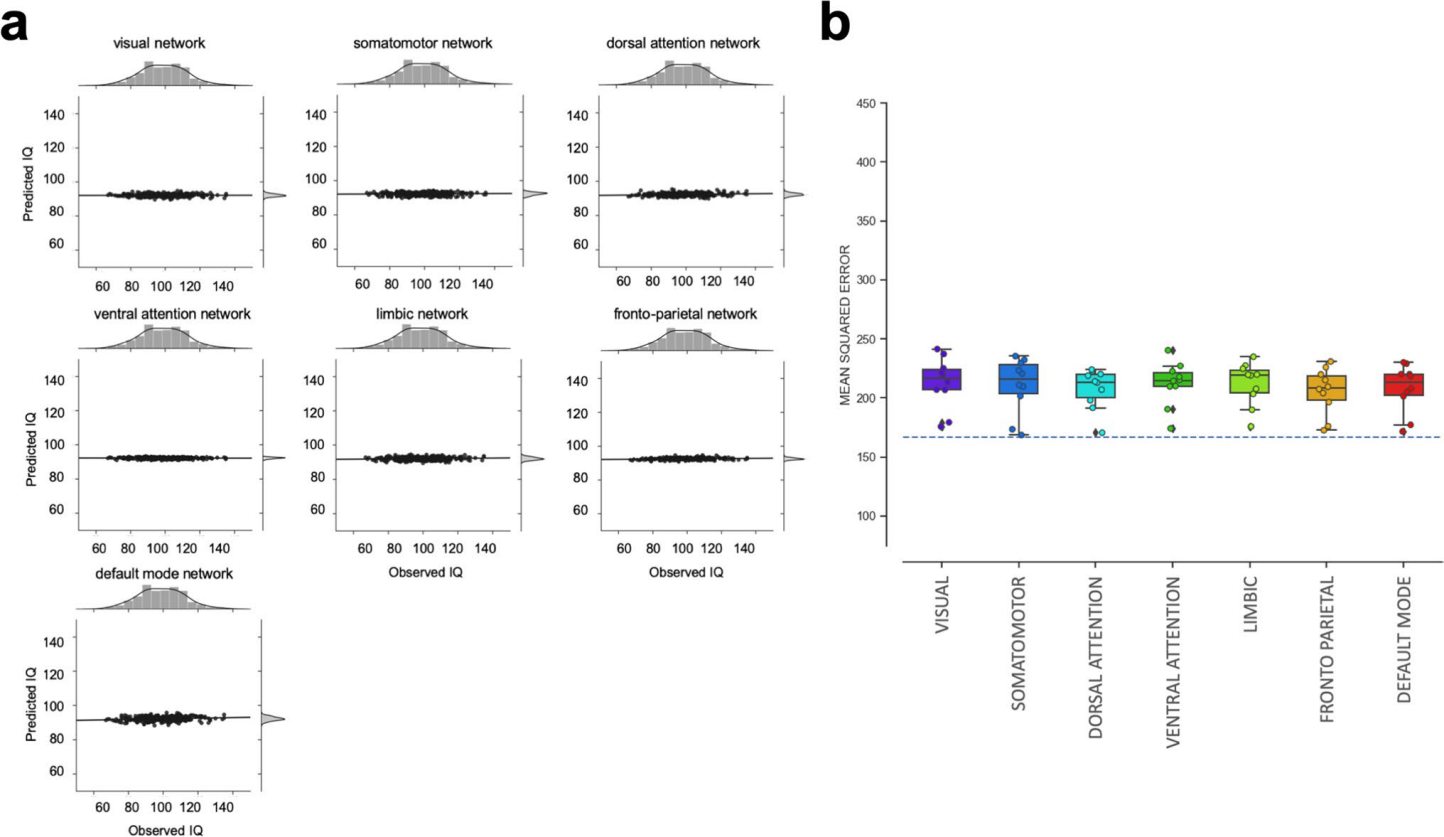
Predictive performance of nine local models based on relative (i.e., TIV-rescaled) gray matter volume and the atlas-based feature construction approach. The nine local models represent the functionally defined brain networks depicted in Fig. 2 (see also "Methods" for further details). **a** Observed (*x-*axis) versus predicted (*y*-axis) FSIQ scores for all 308 participants, separately for each functional network. The gray areas around the regression lines represent the 95%-confidence intervals (determined by bootstrapping) of prediction accuracies. **b** Boxplots illustrating the variability of predictive performance (mean squared error, MSE) across cross-validation folds. The boxes represent the interquartile range, horizontal lines represent the median, and the whiskers extend to points that lie within 1.5 times the interquartile ranges. The dotted line illustrates the performance of a ‘dummy model’ predicting the group-mean IQ of the training sample for every subject of the test sample

Next, we investigated whether intelligence can be predicted from multivariate patterns of relative gray matter volumes within functionally dissociable brain networks (depicted in Fig. 2). In the PCA-based approach, only one out of these nine local models significantly predicted intelligence, i.e., the cerebellum model (MSE = 171, Bonferroni-corrected *p* < 0.0056, see Fig. 5a and Table 1 for predictive performance measures, and Fig. S4 for results of the non-parametric permutation tests; for fold-specific predictive performance, see Fig. 5b and S5). For the cerebellum model, the correlation between predicted and observed scores was *r* = 0.27 (MAE = 10.42, see Table 1, Fig. S3B, RMSE = 13.07, see Table 1, Fig. S3D). Predictive performance of five local models, i.e., of the visual network, the dorsal attention network, the fronto-parietal network, the default-mode network, and of the subcortical network, approached statistical significance but did not pass the threshold when correcting for multiple comparisons (Table 1). In the atlas-informed approach, only the fronto-parietal network significantly predicted intelligence (Bonferroni-corrected *p* < 0.0056, MSE = 205, MAE = 11.49, RMSE = 14.33, *r* = 0.18, see Fig. 6a and Table 1; for results of the non-parametric permutation tests, see Fig. S6; for fold-specific predictive performance, see Figs. 6b and S7). The prediction results based on the dorsal attention network and the default-mode network approached statistical significance but did not pass the threshold when correcting for multiple comparisons (Table 1). Similar to the global model, we also observed that the variance between prediction folds of the local models was markedly reduced when features were built on the basis of a common brain atlas instead of using PCA (compare Figs. 3c, d, 4c, d).

### Influence of brain size on the prediction of intelligence

To assess the effect of total brain size on whole-brain vs. network-specific predictions, all analyses were repeated using voxel-wise absolute gray matter volumes, i.e., without correcting for individual differences in total intracranial volume (TIV). This resulted in statistically significant predictive performance for the global model based on PCA-derived features (MSE = 183, *p* < 0.001, MAE = 10.77, RMSE = 13.50, *r* = 0.24; range of predicted scores: 77–117 IQ points; Table 2, Fig. S8; for results of the non-parametric permutation test, see Fig. S9; for fold-wise predictive performance, see Figs. S10, S11A; for MAE and RMSE see Fig. S12A, C). The atlas-based whole-brain model of absolute gray matter also resulted in statistically significant predictive performance (MSE = 196, *p* < 0.001, MAE = 11.35, RMSE = 14.00, *r* = 0.30; range of predicted scores: 82–104 IQ points; Table 2, Fig. S13; for results of the non-parametric permutation test, see Fig. S14; for fold-wise predictive performance, see Figs. S15, S16A; for MAE and RMSE, see Fig. S17A, C). For both prediction approaches (i.e., PCA based and atlas based), predictive performance appeared improved in terms of the correlation between predicted and observed FSIQ values (*r* = 0.11 vs. *r* = 0.24 and *r* = 0.30, respectively), but not in the MSE (320 and 197 vs. 183 and 196), our primary criterion for evaluating model performance. Consistently, non-parametric permutation tests (two tailed) revealed that there were no significant improvements in predictive performance (MSE) for the global models based on absolute gray matter volume as compared to the global models based on relative gray matter volume (*p* = 0.356, Fig. S18 for PCA-derived features; *p* = 0.750, Fig. S19 for atlas-informed features). Also, MAEs remained above ten IQ points.

**Table 2:** Results of prediction models based on absolute gray matter volume for the PCA-based approach (first row) and for the atlas-informed feature construction method (second row)

	Network size	MSE	*p*_perm_	Range	MAE	RMSE	*r*
Global model	556,694 400	183 196	< 0.001* < 0.001*	136–223 158–218	10.77 11.35	13.50 14.00	0.24 0.30
Local models							
Visual network	52,753 61	202 203	.061 < 0.001*	160–250 164–224	11.74 11.45	14.18 14.22	0.19 0.21
Somatomotor network	46,282 77	245 203	0.836 < 0.001*	150–331 167–225	12.46 11.45	15.52 14.22	0.10 0.19
Dorsal attention network	36,374 46	226 203	0.446 < 0.001*	165–327 163–234	12.43 11.43	15.00 14.22	0.13 0.28
Ventral attention network	32,345 47	206 199	0.018 < 0.001*	142–286 162–220	11.72 11.36	14.25 14.10	0.22 0.22
Limbic network	27,296 26	236 205	0.490 < 0.001*	182–277 174–226	12.49 11.46	15.33 14.30	0.08 0.29
Fronto-parietal network	45,921 52	196 206	0.007 0.002*	153–291 158–233	11.28 11.52	13.96 14.31	0.20 0.27
Default-mode network	71,492 91	210 199	0.068 < 0.001*	171–266 163–228	11.92 11.36	14.48 14.09	0.21 0.29
Subcortical network	20,361 –	225 –	0.267 –	151–268 –	12.14 –	14.95 –	0.16 –
Cerebellum	57,851 –	210 –	0.054 –	149–298 –	11.86 –	14.38 –	0.15 –

Network size is depicted in number of voxels for the PCA-based approach and in number of parcels for the atlas-based feature construction method. Note that in the PCA-based approach the number of features was independent from network size, i.e., features were always 277/278 principal components, whereas in the atlas-based approach the number of features corresponds to the number of parcels, i.e., the network size. Results indicating statistical significance are marked with an asterisk (Bonferroni-corrected for multiple comparisons). *MSE* mean squared error*, p*_*perm*_* p *value of statistical significance computed by non-parametric permutation test, *range* of MSE values resulting from different cross-validation folds, *MAE* mean absolute error in IQ-points, *RMSE* root mean squared error in IQ-points, *r* Pearson’s correlation coefficients between predicted and observed Full-Scale Intelligence Quotient (FSIQ) score. All model fit indices were calculated for each cross-validation fold separately and averaged across folds afterwards

None of the nine local models based on absolute gray matter volumes significantly predicted intelligence using the PCA-based predictive approach (all *p* values larger than the Bonferroni-corrected threshold of *p* = 0.0056). Trend-level significance (i.e., *p* < 0.05 without correcting for multiple comparisons) was observed for the fronto-parietal network and the ventral attention network (Table 2, Fig. S20; for fold-wise predictive performance, see Figs. S10, S11B; for MAE and RMSE, see Fig. S12B, D; for results of the non-parametric permutation test, see Fig. S9). In contrast, when using averaged gray matter values from the Schaefer parcels as features (atlas-based approach), all local models based on absolute gray matter resulted in statistically significant predictions (all *p* values smaller than the Bonferroni-corrected threshold of *p* = 0.0071, Table 2, Fig. S21; for fold-wise predictive performance, see Figs. S15, S16; for MAE and RMSE, see Fig. S17B, D; for results of the non-parametric permutation test, see Fig. S14). None of the differences in predictive performance between local models based on absolute gray matter volumes and local models based on relative gray matter volumes reached statistical significance (PCA-based approach: all *p* values > 0.0056, Fig. S18; atlas-based approach: all *p* values > 0.0071, Fig. S19).

### Additional control analyses

Given that in all cases of significant predictions (see above) the MAE remained rather high (around ten or 11 IQ points), and given that predicted values were clustered in many cases around the sample mean IQ (see Figs. 3, 4, 5, 6), we aimed to assess our model performance against a model that simply uses the group-mean IQ of the training set as predictor for all participants. Such a ‘dummy’ model reached highly comparable model performance (illustrated as additional line in Figs. 3c and 4c for global models and in Figs. 5b and 6b for local models based on relative gray matter volume and in Figs. S11 and S12 for models based on absolute gray matter volume), with a MAE of 10.48 IQ points (MSE = 166.93; RMSE = 12.90).

Finally, to exclude the possibility that our atlas-based results were influenced by the specific choice of a brain atlas, we conducted three additional control analyses: we first repeated both whole-brain analyses (based on relative and on absolute gray matter volumes) with the Schaefer 100 parcellation (Schaefer et al. 2018) to test whether the mere number of features may have had an impact on the prediction results. Second, we used the Shen 264 atlas (Shen et al. 2013) to test the robustness of our effects against another functionally defined parcellation scheme that was built with a different method and based on a different sample than the Schaefer parcellations. Lastly, we conducted the same analyses with the AAL atlas to check whether anatomically derived parcellations would lead to different results in contrast to functionally defined atlases. For relative gray matter volume, likewise to the Schaefer 400 atlas (MSE = 197; *p* < 0.001; see above), also the Schaefer 100 (MSE = 206; *p* < 0.001) and the Shen 264 atlas (MSE = 205; *p* < 0.001) resulted in significant predictions. Only the AAL atlas-based prediction did not reach statistical significance (MSE = 211; *p* = 0.121). For absolute gray matter volume, as for the Schaefer 400 atlas (Schaefer 400: MSE = 196; *p* < 0.001; see above), all atlases yielded significant predictions (Schaefer 100: MSE = 203, *p* < 0.001; Shen 264: MSE = 192, *p* < 0.001; AAL: MSE = 200, *p* < 0.001). The results of these control analyses are illustrated in Supplementary Figures S22–S24, and indicate that atlas-based results are robust against the specific choice of an atlas.

## Discussion

We used two different cross-validated predictive modeling approaches to test whether individual intelligence scores can be predicted from regional brain gray matter volume - beyond the known relationship between intelligence and total brain size (Nave et al. 2018; Pietschnig et al. 2015). Predictive performance of a whole-brain model based on relative gray matter volume was not significantly above chance when using a PCA-based feature construction approach, but reached statistical significance when features were derived from an established functional brain atlas parcellation. Nevertheless, independent of the analysis approach, predictive performance was low in terms of the correlation between predicted and observed IQ scores (*r* = 0.11 in both cases), and the absolute difference between predicted and observed scores varied between 11 and 14 IQ points. The same analyses with absolute gray matter volumes, i.e., without correcting for total brain size, yielded significant prediction in both cases and provided higher correlations between observed and predicted IQ scores (*r* = 0.24 and *r* = 0.30). However, the MAEs remained nearly unchanged (around 11 IQ points). Brain network-specific analyses of relative gray matter volumes resulted in significant predictive performance only for the cerebellum in the PCA-based approach and only for the fronto-parietal network with the atlas-based method. Network-specific prediction from absolute gray matter was not above chance in the PCA-based approach, but provided significant predictions for all networks with the atlas-based method. However, independent of statistical significance, the MAE remained between 11 and 14 IQ points in all network-specific analyses. Critically, and in all cases, the predictive performance in terms of absolute error did not differ in any substantial way from a ‘dummy’ predictive model based on the sample mean - an observation that calls into question the practical value also of those results that reached statistical significance.

To summarize these results, we observed (a) variable results for whole-brain predictive models in terms of statistical significance, with relative gray matter allowing for significant prediction only with the atlas-based feature construction method, while absolute gray matter provided significant predictions with both approaches. We found (b) heterogeneous results with respect to network-specific prediction performance, providing no support for models of gray matter volume and intelligence that focus on only specific regions of the brain. Finally, our results (c) indicate a high absolute error of prediction, which suggests limited practical value of machine learning models predicting general intelligence from patterns of regional gray matter volume. In the following, we will discuss the role of region-specific adaptations of gray matter volume for general intelligence, the separable contributions of relative vs. absolute gray matter volume, conclusions that can be drawn from the network-specific analyses, as well as limitations of the present investigation. Finally, we discuss suggestions and recommendations for future investigations applying predictive modeling approaches to the study of phenotypic variations.

### Predicting intelligence from region-specific variations in relative gray matter volume

Recent evidence suggests that individual intelligence scores can be predicted from functional (resting-state) connectivity (Dubois et al. 2018; Ferguson et al. 2017; Finn et al. 2015; Liu et al. 2018). An earlier study also provided initial evidence for the feasibility of predicting intelligence from brain structure, in that case, based on a combination of various morphometric features (Yang et al. 2013). In the current study, we tested explicitly the predictive performance of one of the most commonly studied structural correlates of intelligence, regional gray matter volume, but found only limited evidence for above-chance prediction of individual intelligence scores when controlling for individual differences in total brain size. This finding is consistent with the results of a very recent machine learning competition which aimed at predicting intelligence in a large cohort of 8669 healthy children from brain structure operationalized by several MRI brain morphological metrics including absolute and relative gray matter volume (ABCD Neurocognitive Prediction Challenge). The final model of that competition did not succeed in significantly predicting intelligence and resulted in only a low correlation of *r* = 0.03 between predicted and observed IQ scores (Mihalik et al. 2019). This study differs from the present work not only regarding the age range of the sample, the broader set of features used for prediction, but also with respect to the to-be-predicted target variable. The intelligence scores provided by the ABCD challenge were estimated from performance in cognitive tasks of the NIH Toolbox Neurocognitive battery (Akshoomoff et al. 2013) but, critically, the resulting scores were residualized with respect to several variables known to be strongly correlated with intelligence, such as highest parental education (e.g., von Stumm and Plomin 2015). Given these differences, it is not clear how directly the two studies can be compared. Nevertheless, they converge in the sense that both studies fail in precisely predicting general intelligence from morphometric patterns of brain anatomy.

The results of the present study also allow for conclusions concerning the heterogeneity of previous structural VBM findings (as also indicated, for example, by the relatively weak meta-analytic effects observed in Basten et al. 2015). Specifically, our present data suggest that some of the previous VBM results (in studies with smaller sample sizes than in the current study) may have been driven primarily by sample-specific variance and may thus not generalize to independent and previously unseen data. Using a predictive rather than an explanatory statistical approach, and by exploring two different feature construction methods, we found no evidence in support of a strong relationship between relative regional gray matter volume and general intelligence. Further, our analyses revealed that even for those three models for which prediction performance was significantly above chance (i.e., the cerebellum model in the PCA-based approach; the whole-brain model and the fronto-parietal model in the atlas-based approach), the average absolute error we would make when predicting intelligence scores of individual persons would be too high for actual applications (i.e., between ten and 14 IQ points).

The practical relevance of an error of around ten to 14 IQ points can be illustrated by considering the impact that a difference of that magnitude may have on critical decisions with long-term consequences, e.g., with respect to whether or not someone is eligible for receiving specific support (like for children with very low or very high cognitive abilities). In this regard, it is also interesting to note that the average effect of 1 year of secondary schooling in adolescence on later IQ has been estimated at between three (Falch and Sandgren Massih 2011) and five (Brinch and Galloway 2012) IQ points. A difference of ten IQ points, thus, may amount to the effect of 2 to 3 years of schooling on IQ, and a prediction error in that range can, therefore, have severe consequences in actual selection or placement decisions.

The visualization of our PCA-based results shows that prediction performance varies across the range of possible IQ scores, with higher prediction accuracies close to the mean and larger errors in the extreme tails of the distribution. This is visible from the confidence interval of prediction accuracy, which is highlighted as a gray area around the regression lines in Fig. 3a, and results primarily from the fact that intelligence is approximately normally distributed in our sample implying that there are more data points available around the mean IQ of 100. The model can thus be ‘better’ trained and generate more accurate predictions within that range - the more instances (of intelligence–gray matter associations) are available within a certain range, the more opportunities the algorithm has to learn these associations and to capture also fine-grained deviations. In contrast, the visualization of atlas-based results (Fig. 4a) indicates a very restricted range of predicted IQ scores (87–99 IQ points) with heavy clustering in a narrow range close to the sample mean. This may result from the fact that the mean represents the maximum-likelihood estimation, which can drive the prediction algorithm and lead to predicted values close to the mean when there is no other relevant pattern found in the data. As in the atlas-based approach, the fold-specific variance is naturally reduced due to common features for all subjects (400 atlas parcels). This pattern becomes especially visible in this method and highlights the limited presence of relevant information in the data after applying the parcellation. The latter point receives further support from our observation of comparable predictive performance when strictly using the group-mean IQ as predicted score for all participants (see “Additional control analyses”: ‘dummy model’). Thus, the difference in the statistical significance of prediction results obtained for the atlas-based prediction models in contrast to the PCA-based models on relative gray matter volumes may primarily result from an over-representation of IQ values around the sample mean (due to normally distributed IQ scores) that, due to the algorithm’s tendency to use the sample mean as best predictor when no other relevant information is available, lead to reduced variance between folds and thus an increased likelihood of statistical significance.

However, it is important to note that this does not mean that the significance of results is artificial, but that PCA- and atlas-based approach are differentially dependent on fold-specific variability. It may thus be more a theoretical decision whether one prefers an approach that relies purely on the given input data (PCA) or an approach that is informed by domain-specific knowledge. For instance, in cases where no prior assumptions about the underlying data structure exist or where the (arbitrary) choice of a specific brain atlas should be prevented, a purely data-driven approach would represent the preferred method. However, a purely data-driven approach can also increase the generalization error and induce fold-specific variance (since the model is fitted to the training set and might overfit). This can especially be the case when samples are small (< 1000) in relation to the high-dimensional input data, as it is mostly the case in human neuroimaging studies. In contrast, a domain knowledge-based approach introduces a priori assumptions (that may or may not be correct) and will therefore less likely overfit to the training data. This can reduce fold-specific variance and minimize the generalization error, but respective prediction models can only generalize to data of the same structure, i.e., MRI data that are preprocessed in the same way and parcellated with the same atlas. This trade-off between generalizability and accuracy has to be considered thoroughly when selecting the feature construction method.

Additionally, the pattern of our results suggests that test statistics like the MAE, which can be interpreted in terms of absolute IQ points, are of obvious informative value. To the best of our knowledge, such measures have not been considered as criteria for model evaluation in previous studies that reported successful prediction of intelligence from task-induced activation (Sripada et al. 2018) or intrinsic connectivity (Dubois et al. 2018; Ferguson et al. 2017; Finn et al. 2015; Liu et al. 2018), which impedes the direct comparability of our results to these former studies. However, a similar restriction of the variance of predicted intelligence around the mean, as observed in our study, is also present, for example, in the significant prediction results of Finn et al. (2015; see their Fig. 5a, c) and Dubois et al. (2018; see their Fig. 3a). Error measures like the MSE or the MAE yield important additional insights into the practical relevance of prediction-based neuroimaging studies, and we would, therefore, advocate their use in future studies.

### Relative vs. absolute gray matter volume and their relevance for general intelligence

In contrast to the mixed results obtained in respect to the whole-brain patterns of relative gray matter volume, whole-brain patterns of absolute gray matter volume provided statistically significant predictions of intelligence irrespective of the specific feature construction method - albeit again with a rather high MAE of (around 11 IQ points) and with a highly restricted ranged of predicted values in the atlas-based models. This may suggest that regional differences in gray matter volume do contribute some but not much information beyond total brain size. Importantly, however, the differences in predictive performance between models based on relative vs. absolute gray matter volume were not statistically significant - neither for the global models nor for any of the local models and neither in the PCA-based nor in the atlas-based approach, rendering such conclusions preliminary. Nevertheless, our result underscores the importance of differentiating thoroughly between relative and absolute gray matter and to compare respective effects, particularly given that the variable of interest (IQ) is significantly related to brain size (McDaniel et al. 2005; Nave et al. 2018; Pietschnig et al. 2015). It is not absolutely clear what neurobiological characteristics are primarily reflected in gray matter probability maps as derived from VBM: More cell bodies, neutrophil, glia cells, synapses, and capillaries all seem to be related to higher gray matter values, but also more cortex folding and thicker gray matter can contribute to high gray matter indices (Mechelli et al. 2005). Most often, however, gray matter values are interpreted as reflecting the total amount of neuronal packing within a certain region, i.e., an approximation of neuron number (Gaser and Kurth 2018). Variations in total brain size are thus likely to reflect individual differences in total neuron numbers (e.g., Leuba et al. 1994; Pakkenberg and Gundersen 1997) and positive associations with intelligence are typically interpreted as indicating more computational processing power due to larger neural capacities (e.g., in Genç et al. 2018). The results of our analyses of absolute gray matter volumes are well in line with this proposal and extend it in suggesting that this positive association, i.e., between higher intelligence and more computational power due to more neurons, may exist in all functional brain networks. In contrast, relative gray matter volume reflects local deviations in neuron number that goes beyond the neuron number that one would expect for a given region on the basis of an individual’s brain size. The low predictive performance of relative gray matter models observed in our study suggests only a minor influence of these deviations (beyond brain size) on individual differences in intelligence. Overall, our results are more in support of theories proposing intelligence as a result of a global processing advantage, rather than theories of intelligence focusing on region-specific gray matter characteristics.

### Differences in predictive performance between functional brain networks

Our results of the network-specific (local) analyses of relative gray matter volume demonstrate that even when restricting the number of features by separately modeling distinct functional brain networks, only two sub-systems could predict intelligence significantly above chance, i.e., the cerebellum in the PCA-based approach and the fronto-parietal network in the atlas-based method. The observation that frontal and parietal brain regions are more closely related to individual differences in intelligence than other regions is well in line with previous observations and neurocognitive theories of intelligence (e.g., P-FIT model, Basten et al. 2015; Jung and Haier 2007; Multiple-Demand System, Duncan 2010), while the cerebellum has typically not been considered as relevant for individual differences in intelligence. Contrasting these network-specific differences in the predictability of intelligence from relative gray matter volume, the local models based on absolute gray matter did not differ between each other in respect to their significance: While none of the network models approached significance in the PCA-based approach, all models provided above-chance predictions with the atlas-based method. Critically, however, in all local models, the MAE was comparably high (i.e., between ten and 12 IQ points). As already discussed for the global models, this observation limits the impact of network-specific differences in gray matter volume for the understanding and prediction of general intelligence.

The currently available evidence from prediction-based studies, thus, seems to suggest that brain function (i.e., resting-state functional connectivity or task-induced brain activation) may be more important than brain structure in determining individual differences in general cognitive ability - at least when operationalizing brain structure exclusively as regional gray matter volume differences. Highest prediction accuracies have so far been reported with respect to intrinsic functional connectivity, i.e., correlated neural activation patterns measured in the absence of any task demand (Dubois et al. 2018; Ferguson et al. 2017; Finn et al. 2015; but note also Greene et al. 2018 for task-based prediction models). As the organization of intrinsic brain networks is assumed to be closely related to the underlying anatomical connectivity backbone, i.e., the strongest structural connections between different brain regions (Greicius et al. 2009), we speculate that measures of structural connectivity (as assessed, e.g., with diffusion tensor imaging) may allow for a more accurate prediction of general intelligence than volumetric indices of regional gray matter volume (for correlative support of this assumption, see, e.g., Genç et al. 2018). On the other hand, intelligence has also been linked to other regionally specific morphometric properties of the brain such as cortical surface area (e.g., Schnack et al. 2014), gyrification (e.g., Gregory et al. 2016), or cortical thickness (e.g., Karama et al. 2011). Future predictive work, in our view, should thus aim at more strongly integrating the different functional and neuroanatomical characteristics of the brain, to better understand their respective roles for general cognitive abilities.

### Limitations

The machine learning pipeline of the present study used a support vector regression with a linear kernel. This limited our analyses to the detection of linear relationships between intelligence and brain structure. Although this approach is one of the most widely used in the field of neuroimaging (for review, see Lemm et al. 2011; Pereira et al. 2009), the possible existence of non-linear associations cannot be excluded. However, our selection of this approach was driven a) by computational feasibility (the reported analyses took an equivalent of ~ 36,000 h of computation time with 2 CPU kernels and 5 GB RAM; non-linear analyses would take substantially longer) and b) by our aim of reaching highest comparability with previous correlative analyses on brain structure and intelligence (from explanatory studies, see above).

Second, our results revealed considerable variance in predictive performance across the ten folds of the cross-validation procedure, despite our efforts to homogenize the distributions of the target variable (IQ) between folds. This was particularly severe in the PCA-based approach, but also obvious in models that relied on the atlas-informed feature construction method. A systematic investigation of the heterogeneity in prediction performance across folds could be achieved, e.g., by repeating all analyses 100 times and then examining differences between resulting distributions of prediction accuracies. This is, however, at present not computationally feasible. To the best of our knowledge, the variability of results across folds has not been addressed in detail by previous machine learning-based neuroimaging investigations and our study is one of the first to illustrate fold-specific predictive performances at all. In our opinion, this observation deserves closer consideration in future research and we, therefore, recommend reporting (in addition to overall predictive performance) always also fold-specific measures of predictive performance.

Finally, for predictive modeling approaches like the one used in the present study, the use of many data points is essential to train the prediction models sufficiently and to gain stable prediction weights. Of note, it has been observed that prediction accuracies increase as sample size decreases (Varoquaux 2017), suggesting the presence of unrealistically exaggerated (and thus invalid) prediction accuracies in studies using small samples. Although our sample size can be considered large relative to other prediction studies from recent years (for comparison of prediction-based neuroimaging studies, see, e.g., Arbabshirani et al. 2017; Poldrack et al. 2020), it nevertheless appears small given the dimensionality of the original feature space (i.e., the number of voxels in the brain). We thus propose that future work should strive to further increase sample sizes, for example by combining data from different sources (as is done in genetics; e.g., Savage et al. 2018).

### Methodological implications and recommendations for future studies

In light of the results presented in this work, we would like to summarize methodological insights that may be valuable to consider in future predictive studies, within the field of intelligence research but also more generally in individual differences-focused predictive modeling investigations. First, whenever cross-validation is used to assess the performance and generalizability of the predictive model, some measure or visualization of the variance across folds should be reported. Second, predictive variance within folds should be visualized using scatter plots so that the range of the predicted scores becomes transparent. This is especially important for detecting cases in which predicted and true scores correlate highly despite a restricted range of predicted values, indicating poor practical utility of those predictions. Third, pertaining to the same point, measures of the absolute difference between predicted and true values such as RMSE or MAE should be used in addition to the correlation between predicted and observed scores or explained variance. These metrics quantify the error in units of the original scale and are therefore of high value for interpretation. Correlations, on the other hand, are insensitive to the scaling of the original measures, which can lead to high correlations between predicted and observed scores despite considerable differences in their absolute values (see also Poldrack et al. 2020, for an in-depth discussion). Fourth, a comparison of model performance indices with those obtained by a non-informative, ‘baseline’ solution (such as predicting the mean of the training set for all subjects of the test set) can help in interpreting resulting performance measures. Fifth, our results indicate that purely data-driven methods of feature construction (such as PCA) can lead to different results than methods using features informed by domain-specific knowledge (such as using a functionally defined brain atlas). Similar variations in results have been observed for the application of different algorithms and other data transformations (Wolpert and Macready 1997). We therefore recommend to explore the influence that variations in analysis pipelines, such as different feature construction methods, may have on the results, and to report respective observations in detail to achieve a more realistic understanding about the robustness and generalizability of respective findings. In subsequent stages of a research program, such parameters should be defined prior to the data analysis or optimized in a purely data-driven way (within a further inner cross-validation loop), to reduce researcher degrees of freedom and to move from exploratory to more confirmatory research.

## Concluding remarks

The current study used a machine learning-based predictive modeling approach to test whether individual intelligence scores can be predicted from spatially highly resolved (i.e., voxel wise) patterns of regional gray matter volume. When analyzing relative gray matter volumes, i.e., independent of total brain size, predictive performance for the whole-brain model was generally low and reached statistical significance only with a domain knowledge-based feature construction approach (using a common brain atlas) but not with a purely data-driven method (PCA). In contrast, absolute gray matter volume (uncorrected for brain size) allowed for significant predictions of individual intelligence scores with both feature construction approaches. Importantly, the absolute error was relatively high (greater than ten IQ points) and the range of predicted IQ scores was markedly restricted around the sample mean, limiting the practical value of these findings. Brain network-specific analyses of gray matter volume highlight the role of the fronto-parietal network and the cerebellum, but could not reduce the MAE in comparison to the global models. Overall, our results suggest (a) that absolute gray matter volume is a significant predictor of individual differences in intelligence and that this generalizes across functional brain networks, (b) that regional differences that go beyond the influence of brain size (relative gray matter volume) contribute some but not much additional information to this prediction, and (c) that the empirical evidence in favor of region or network-specific gray matter models of intelligence is limited. This supports the proposal that intelligence may be related to global more than region-specific variations in gray matter volume. The difference between our result and earlier reports of significant correlative associations between intelligence and gray matter volume underscores the importance of predictive as opposed to explanatory approaches in the cognitive neurosciences. To be able to unequivocally establish brain–behavior associations, individual difference-oriented neuroimaging studies should strive for true out-of-sample prediction in independent data.

## Electronic supplementary material

Below is the link to the electronic supplementary material.

Supplementary file1 (PDF 9549 kb)
