# Supplementary material for: Predicting intelligence from brain gray matter volume
Source: Brain Struct Funct. 2020 Jul 21;225(7):2111–29. doi: 10.1007/s00429-020-02113-7 (PMC7473979; doi:10.1007/s00429-020-02113-7)
Supplement: Supplementary file 1 — Supplementary file1 (PDF 9549 kb) [file 429_2020_2113_MOESM1_ESM.pdf]

Predicting Intelligence from Brain Gray Matter Volume

Kirsten Hilger[1,2,3*+], Nils R. Winter[4+], Ramona Leenings[4], Jona Sassenhagen[1], Tim Hahn[4], Ulrike Basten[1], Christian J. Fiebach[1,3,5]

[1] Department of Psychology, Goethe University Frankfurt, Frankfurt am Main, Germany
[2] Department of Psychology, Julius Maximilian University Würzburg, Würzburg, Germany
[3] IDeA Center for Individual Development and Adaptive Education, Frankfurt am Main, Germany
[4] Institute of Translational Psychiatry, University Hospital Münster, Münster, Germany
[5] Brain Imaging Center, Goethe University Frankfurt, Frankfurt am Main, Germany

[+] these authors share first authorship

SUPPLEMENTARY MATERIAL

* Kirsten Hilger (corresponding author)
KH is now at University Wuerzburg:
Department of Psychology I
Marcusstr. 9-11
D-97070 Würzburg

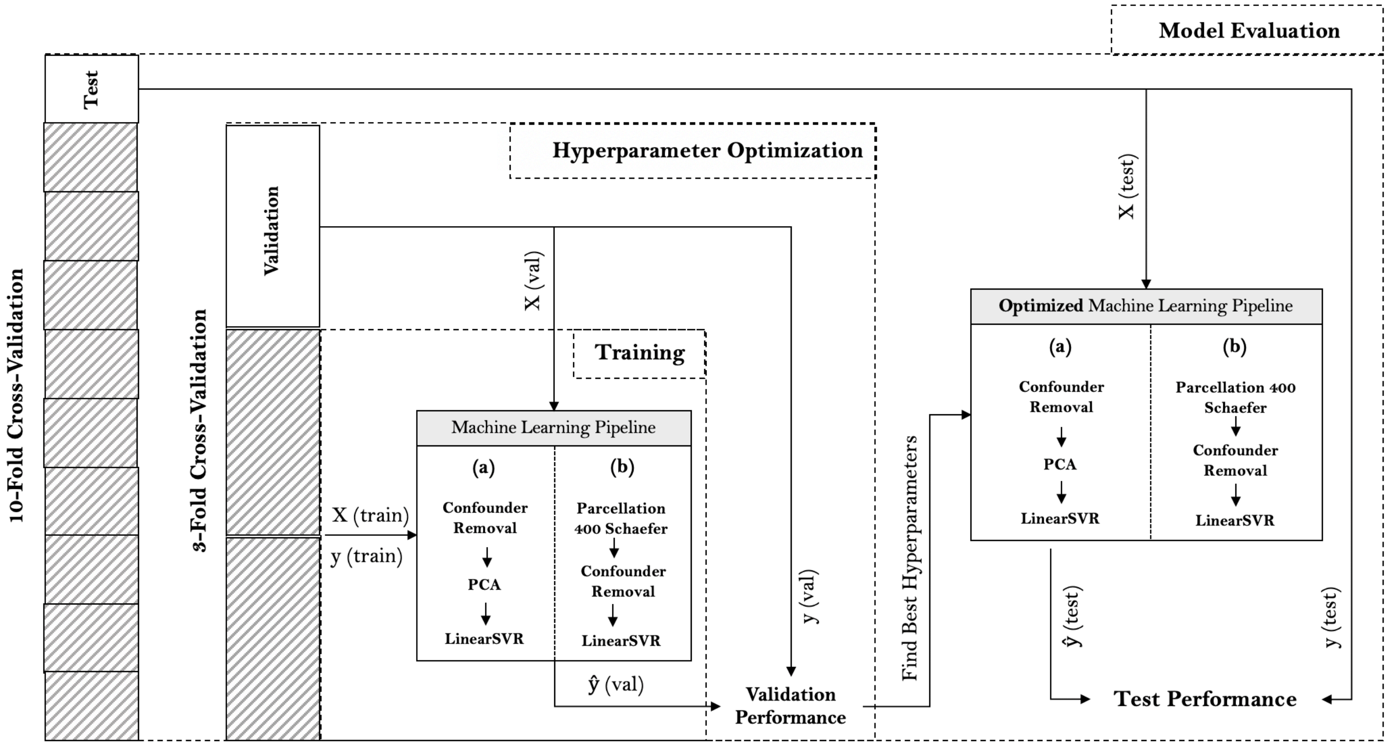

Supplementary Fig. S1 Schematic overview of the machine learning pipeline and the cross-validation approach. First, the data are split into 10 folds as part of the 10-fold cross-validation used to estimate the overall model performance (outer loop). 90% of the data (striped boxes on the far left) is used to optimize the hyperparameters of the SVR machine learning model. On these data, we used a further 3-fold cross-validation procedure to obtain mean validation performances for 50 different hyperparameter configurations (inner loop). We then chose, within each fold of the outer loop, the configuration with the lowest mean squared error (*MSE*) and retrained it on the complete training set (90%) to obtain the optimized machine learning model. This optimized model was then used to generate a prediction for the test set (10%) and to determine the final prediction performance of the respective fold as the mean squared error (*MSE)* between predicted and observed IQ scores. Finally, we computed the mean over all 10 fold-specific test performance measures (fold-specific *MSE*s) to generate the final model performance scores. In addition to the mean *MSE*, we also computed the mean absolute error (*MAE*), the root mean squared error (*RMSE*), and determined the Pearson's correlation coefficient (*r*) between observed and predicted IQ scores. *PCA* principal component analysis, *SVR* support vector regression, *MSE* mean squared error

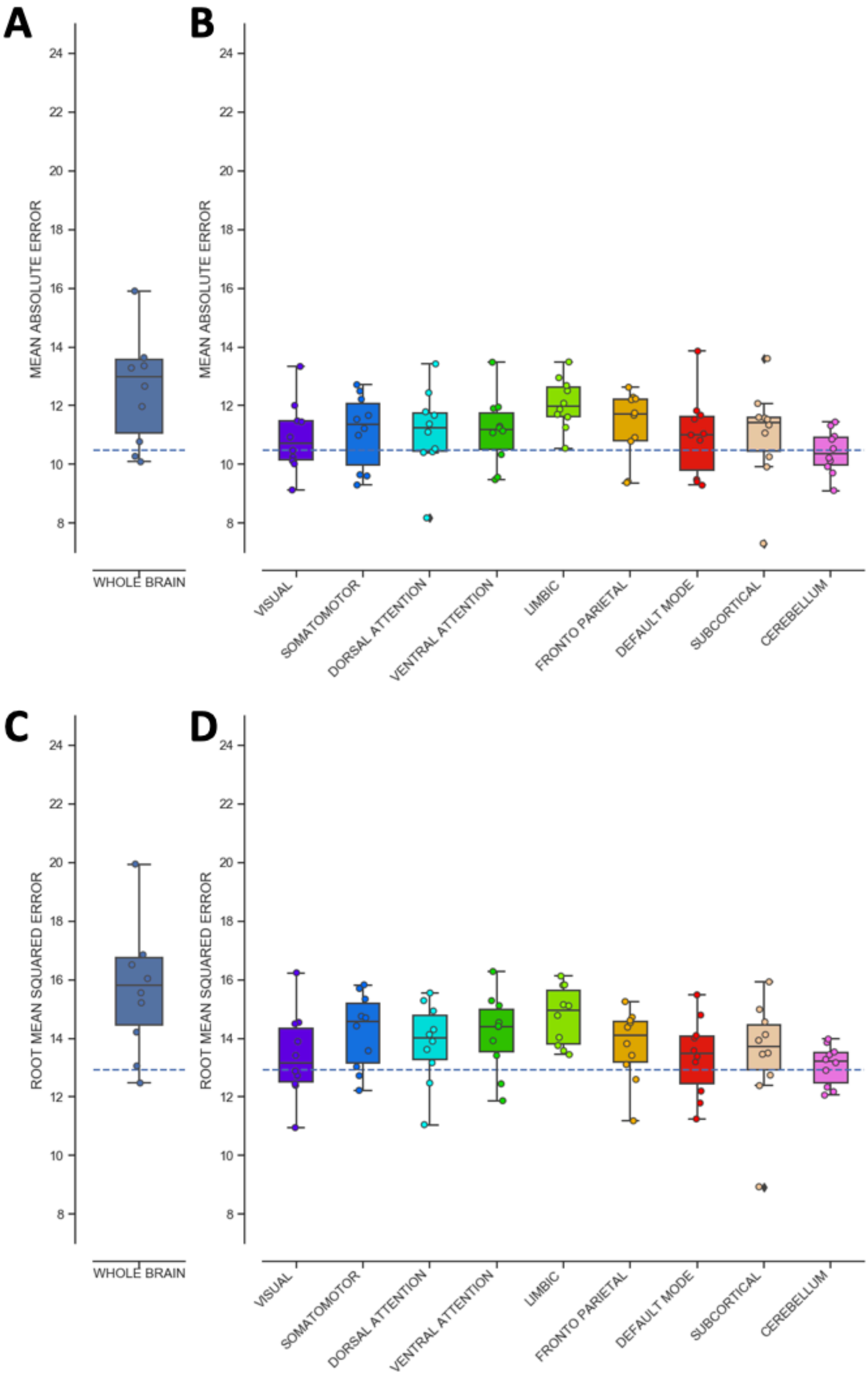

Supplementary Fig. S2 Mean absolute error (*MAE*) and root mean squared error (*RMSE*) results for prediction models based on *relative* gray matter volume and the PCA-based feature construction approach. Boxplots illustrating the variability of predictive performance (upper row: *MAE*; lower row: *RMSE*) across cross-validation folds for the *global* model (**A,C**) and the nine functional brain networks separately (**B,D**). The boxes represent the interquartile range, horizontal lines represent the median, and the whiskers extend to points that lie within 1.5 times the interquartile ranges. The blue dotted line illustrates the performance of a 'dummy model' predicting the group-mean IQ of the training sample for every subject of the test sample

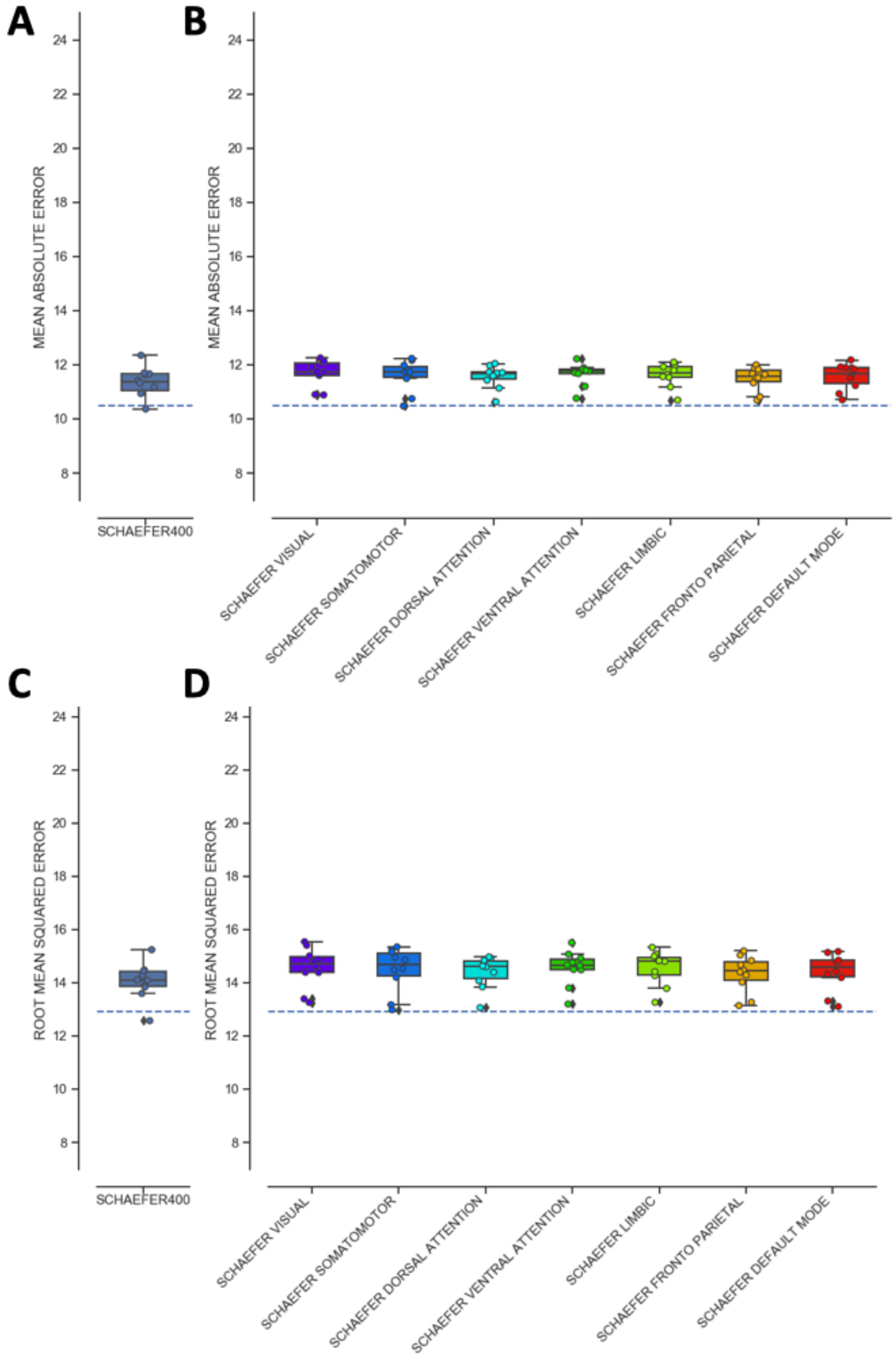

Supplementary Fig. S3 Mean absolute error (*MAE*) and root mean squared error (*RMSE*) results for prediction models based on *relative* gray matter volume and the PCA-based feature construction approach. Boxplots illustrating the variability of predictive performance (upper row: *MAE*; lower row: *RMSE*) across cross-validation folds for the *global* model (**A,C**) and the seven functional brain networks separately (**B,D**). The boxes represent the interquartile range, horizontal lines represent the median, and the whiskers extend to points that lie within 1.5 times the interquartile ranges. The blue dotted line illustrates the performance of a 'dummy model' predicting the group-mean IQ of the training sample for every subject of the test sample

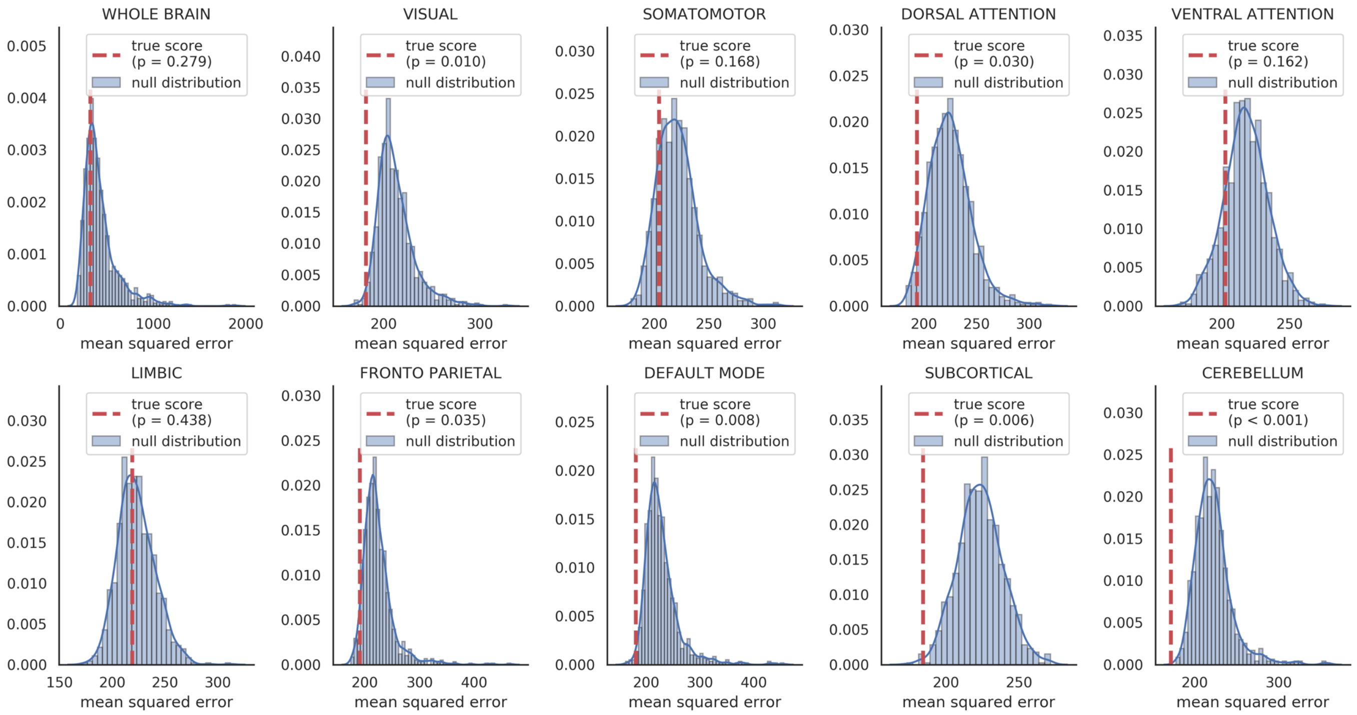

Supplementary Fig. S4 Results of the non-parametric permutation tests for the *global* (whole brain) prediction model and all nine functional network models (*local* models) based on *relative* gray matter volume and the PCA-based feature construction approach. The histograms show the predictive performance given surrogate-null data, i.e., the distribution of the test statistic (mean squared error, *MSE*) based on permuted data ($N = 1{,}000$ permutations; blue line: KDE smoothing) in relation to the predictive performance (*MSE*) based on the observed (i.e., non-permuted) data (red vertical line). If the *MSE* of the observed data had occurred in the extreme tails of the surrogate/permuted data, the prediction result from the machine learning pipeline would have been highly unlikely to be generated by chance, and thus considered significant. The *p*-values resulted from summing up the times in which model performance based on the true targets was lower than model performance based on the permuted targets and dividing this number by the number of permutations. Thus, *p*-values correspond to the percentile position of the observed *MSE* in the distribution of surrogate-null values. The left upper panel repeats the plot shown in Fig. 3b in the main text

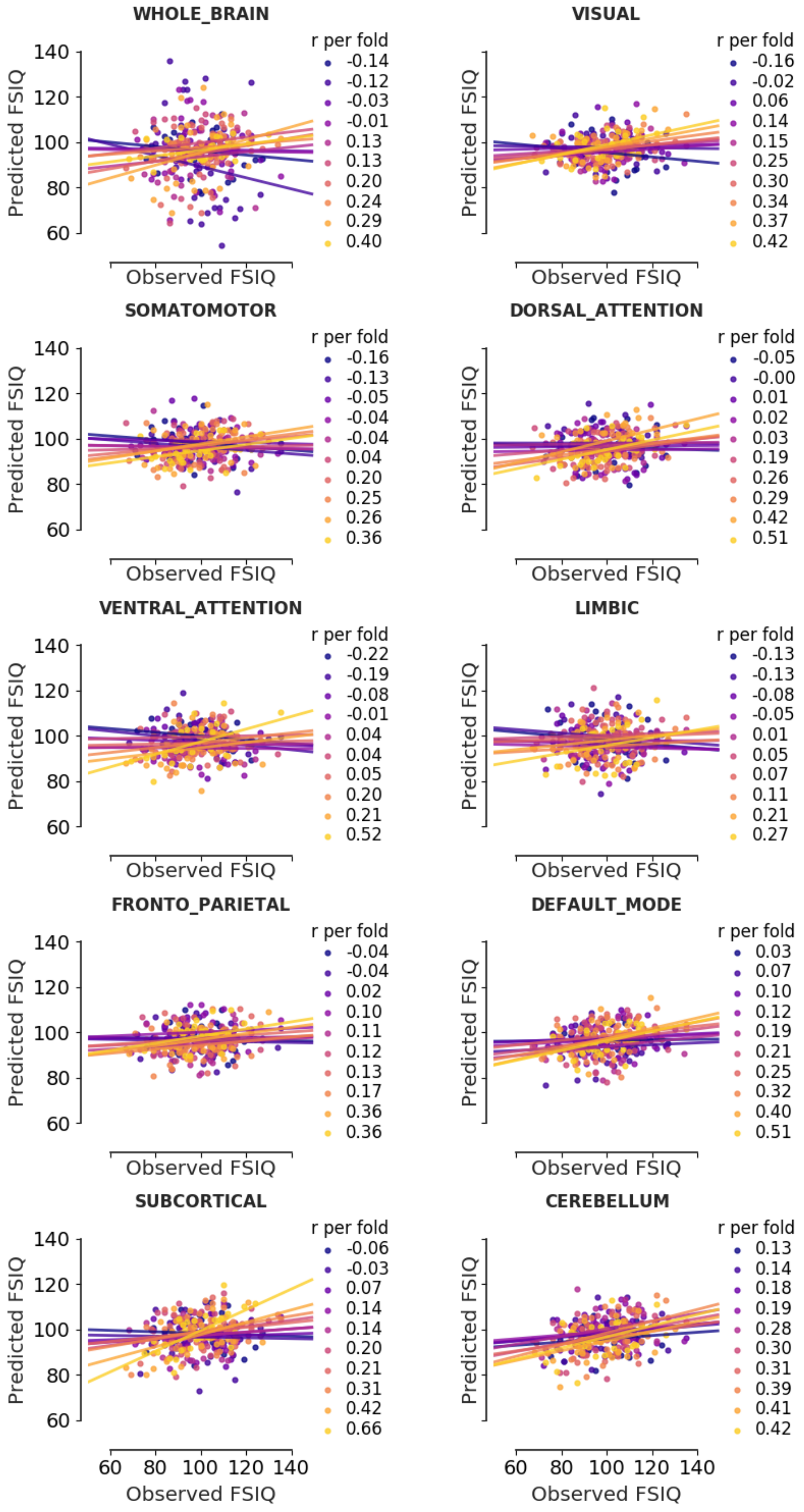

Supplementary Fig. S5 Fold-wise predictive performance for the *global* (whole brain) prediction model and all nine functional network models (*local* models) based on *relative* gray matter volume and the PCA-based feature construction approach. Observed (x-axis) versus predicted (y-axis) full scale intelligence quotient (FSIQ) scores for all 308 participants based on all *relative* gray matter volume values of the brain. Predictions of each cross-validation fold and the corresponding approximated linear regression slopes are highlighted in different colors. The left upper panel repeats the plot shown in Fig. 3d in the main text. *r* Pearson's correlation coefficients between predicted and observed FSIQ score

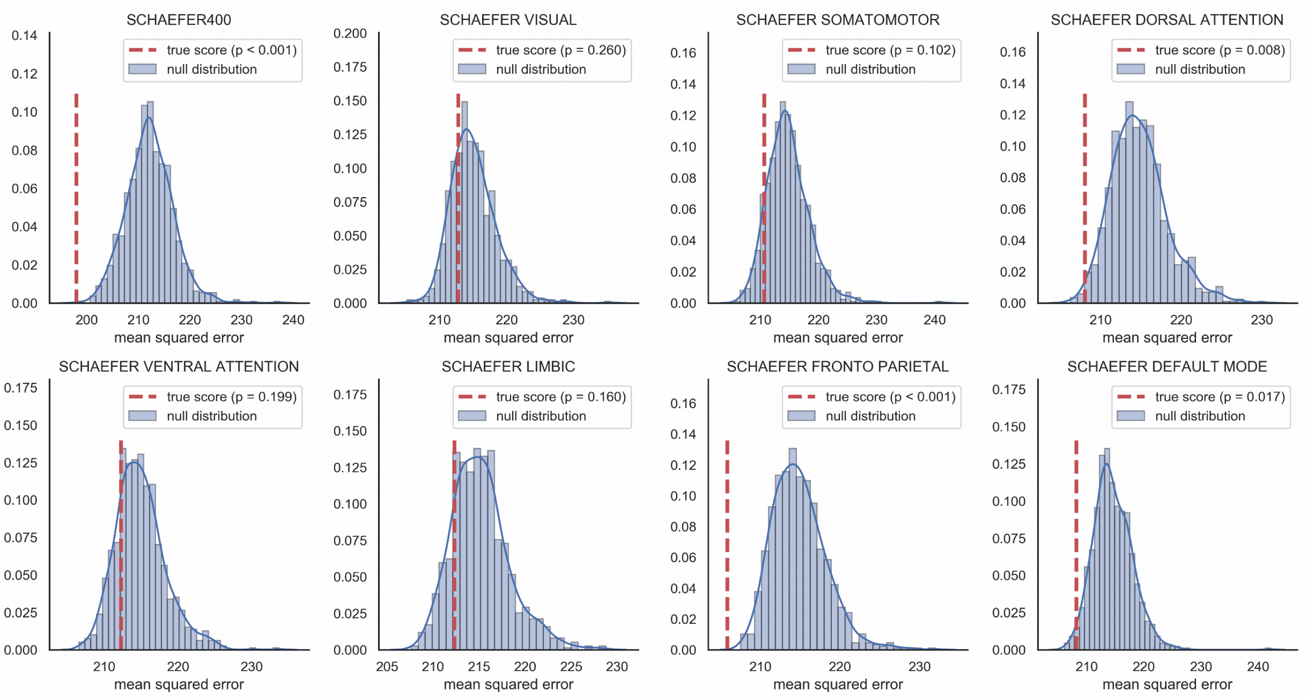

Supplementary Fig. S6 Results of the non-parametric permutation tests for the *global* (whole brain) prediction model and all seven functional network models (*local* models) based on *relative* grey matter and the atlas-based feature construction approach. The histograms show the predictive performance given surrogate-null data, i.e., the distribution of the test statistic (mean squared error, *MSE*) based on permuted data ($N = 1{,}000$ permutations; blue line: KDE smoothing) in relation to the predictive performance (*MSE*) based on the observed (i.e., non-permuted) data (red vertical line). If the *MSE* of the observed data had occurred in the extreme tails of the surrogate/permuted data, the prediction result from the machine learning pipeline would have been highly unlikely to be generated by chance, and thus considered significant. The *p*-values resulted from summing up the times in which model performance based on the true targets was lower than model performance based on the permuted targets and dividing this number by the number of permutations. Thus, *p*-values correspond to the percentile position of the observed *MSE* in the distribution of surrogate-null values. The left upper panel repeats the plot shown in Fig. 4b in the main text

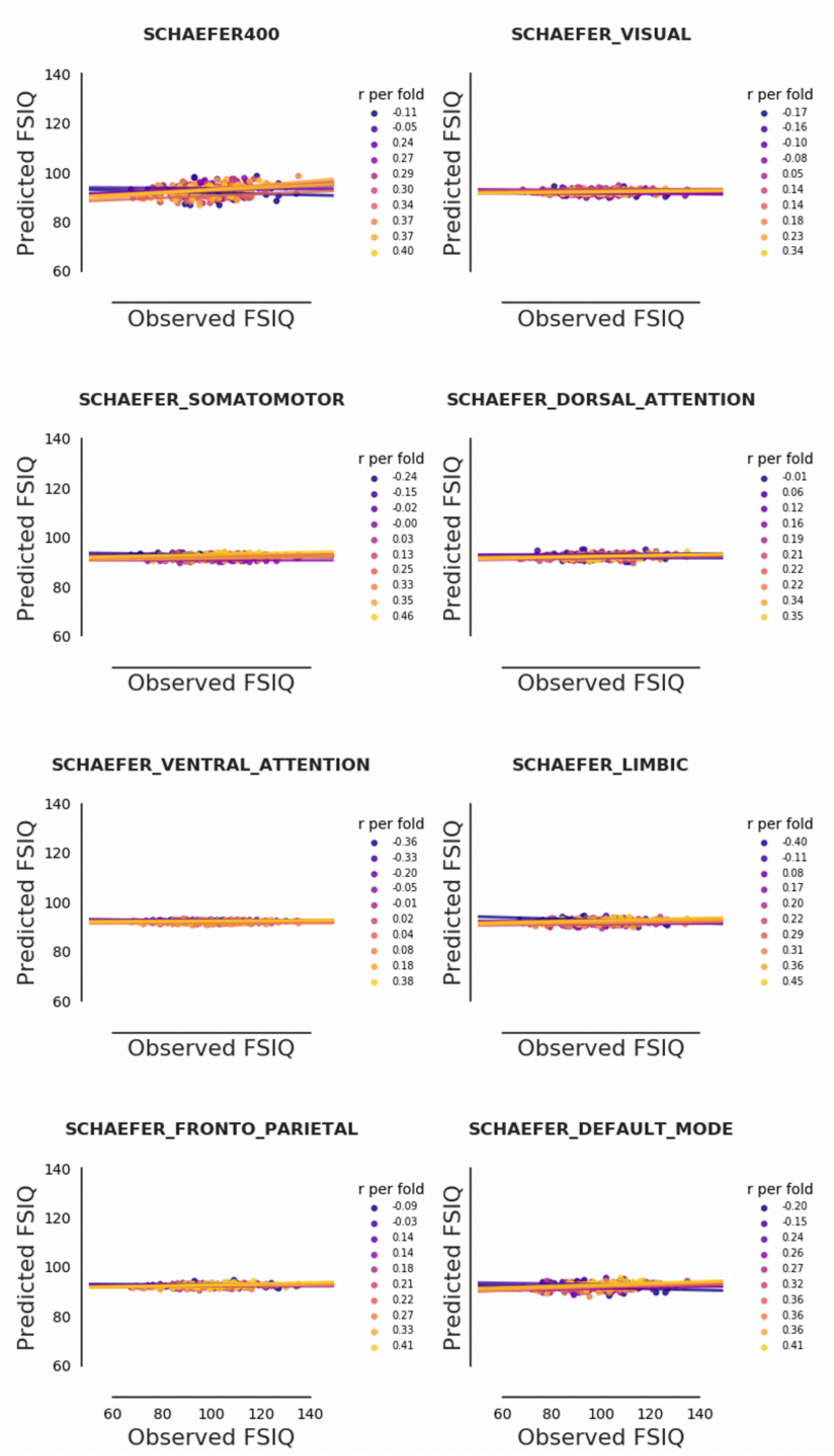

Supplementary Fig. S7 Fold-wise predictive performance for the *global* (whole brain) prediction model and all seven functional network models (*local* models) based on *relative* gray matter volume and the atlas-based feature construction approach. Observed (x-axis) versus predicted (y-axis) full scale intelligence quotient (FSIQ) scores for all 308 participants based on all averaged *relative* gray matter volume values of the brain using the Schaefer parcellation. Predictions of each cross-validation fold and the corresponding approximated linear regression slopes are highlighted in different colors. The left upper panel repeats the plot shown in Fig. 4d in the main text. *r* Pearson's correlation coefficients between predicted and observed FSIQ score

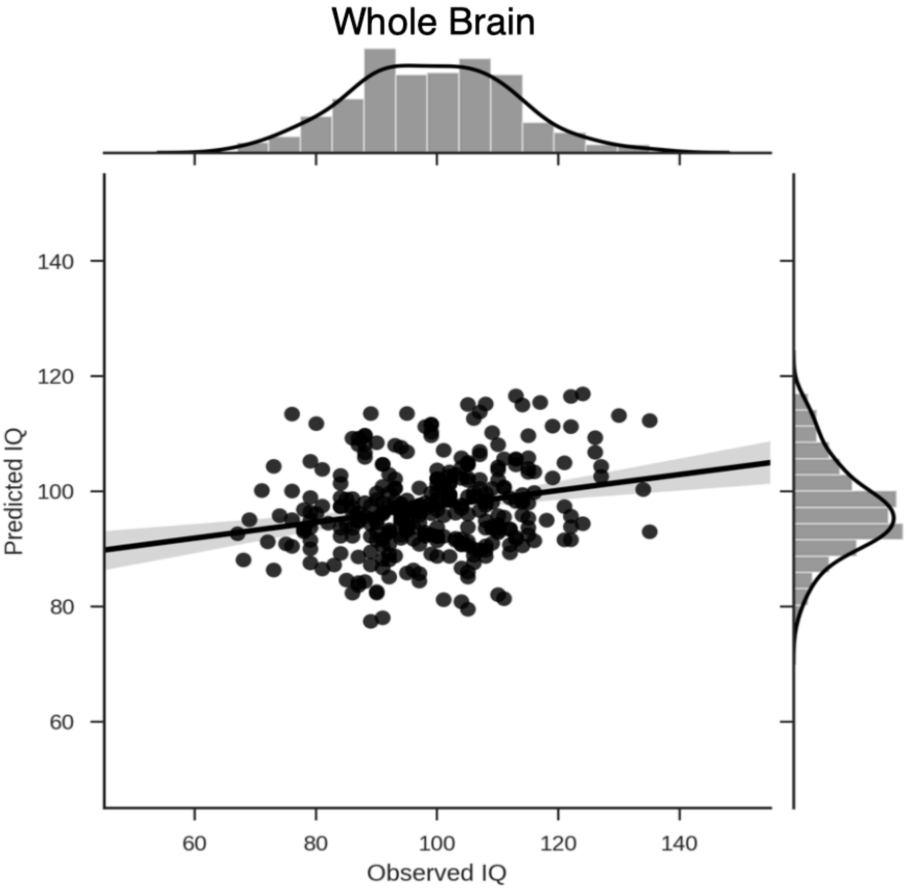

Supplementary Fig. S8 Predictive performance of the *global* (whole brain) model based on *absolute* gray matter volume, i.e., without correction for individual differences in brain size, and the PCA-based feature construction approach. Observed (*x*-axis) versus predicted (*y*-axis) full scale intelligence quotient (FSIQ) scores for all 308 participants. The gray area around the regression line represents the 95%-confidence interval (determined by bootstrapping) of prediction accuracy

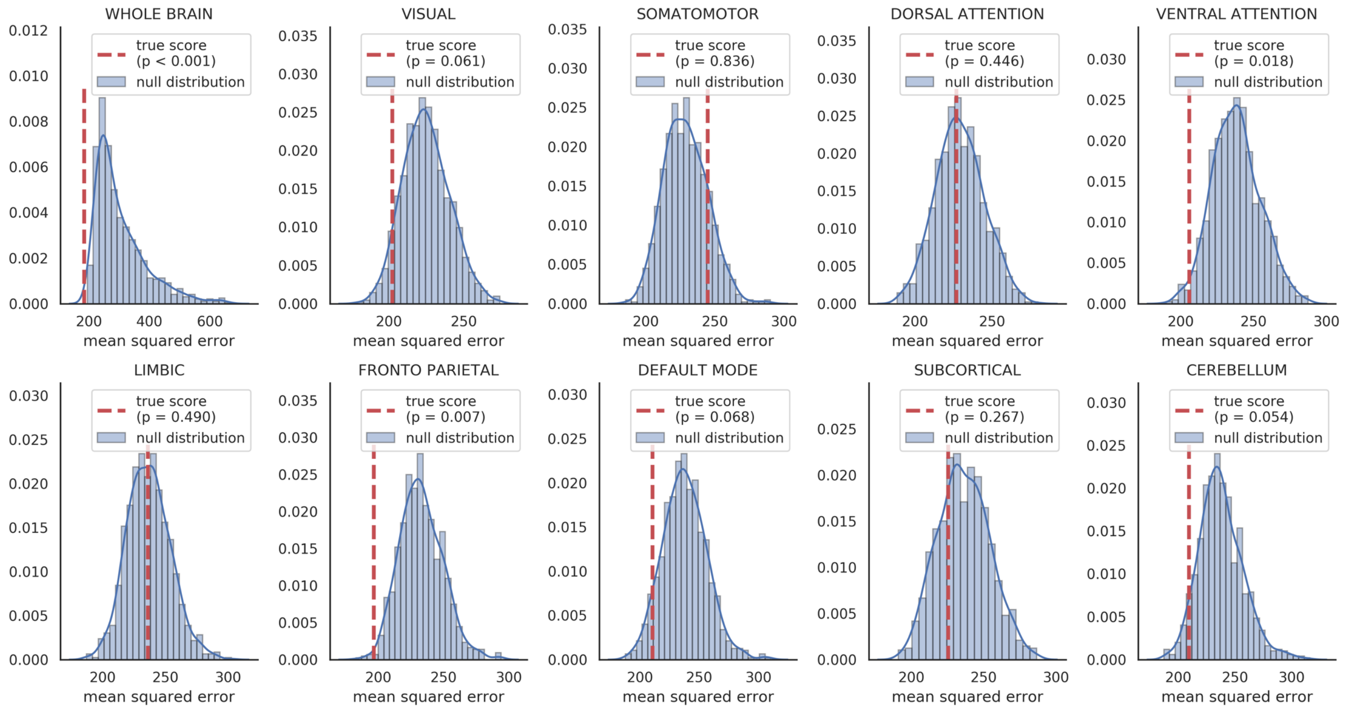

Supplementary Fig. S9 Results of the non-parametric permutation tests for the *global* (whole brain) prediction model and all nine functional network models (*local* models) based on *absolute* gray matter volume, i.e., without correction for individual differences in brain size, and the PCA-based feature construction approach. The histogram shows the predictive performance given surrogate-null data, i.e., the distribution of the test statistic (mean squared error, *MSE*) based on permuted data ($N = 1{,}000$ permutations, KDE smoothing: blue line) in relation to the predictive performance (*MSE*) based on the observed (non-permuted) data (red vertical line). If the *MSE* of the observed data had occurred in the extreme tails of the surrogate/permuted data, the prediction result from the machine learning pipeline would have been highly unlikely to be generated by chance, and thus considered significant. The *p*-values resulted from summing of the times in which model performance based on the true targets was lower than model performance based on the permuted targets and dividing this number by the number of permutations, i.e., 1,000. *p*-values correspond to the percentile position of the observed *MSE* in the distribution of surrogate-null values

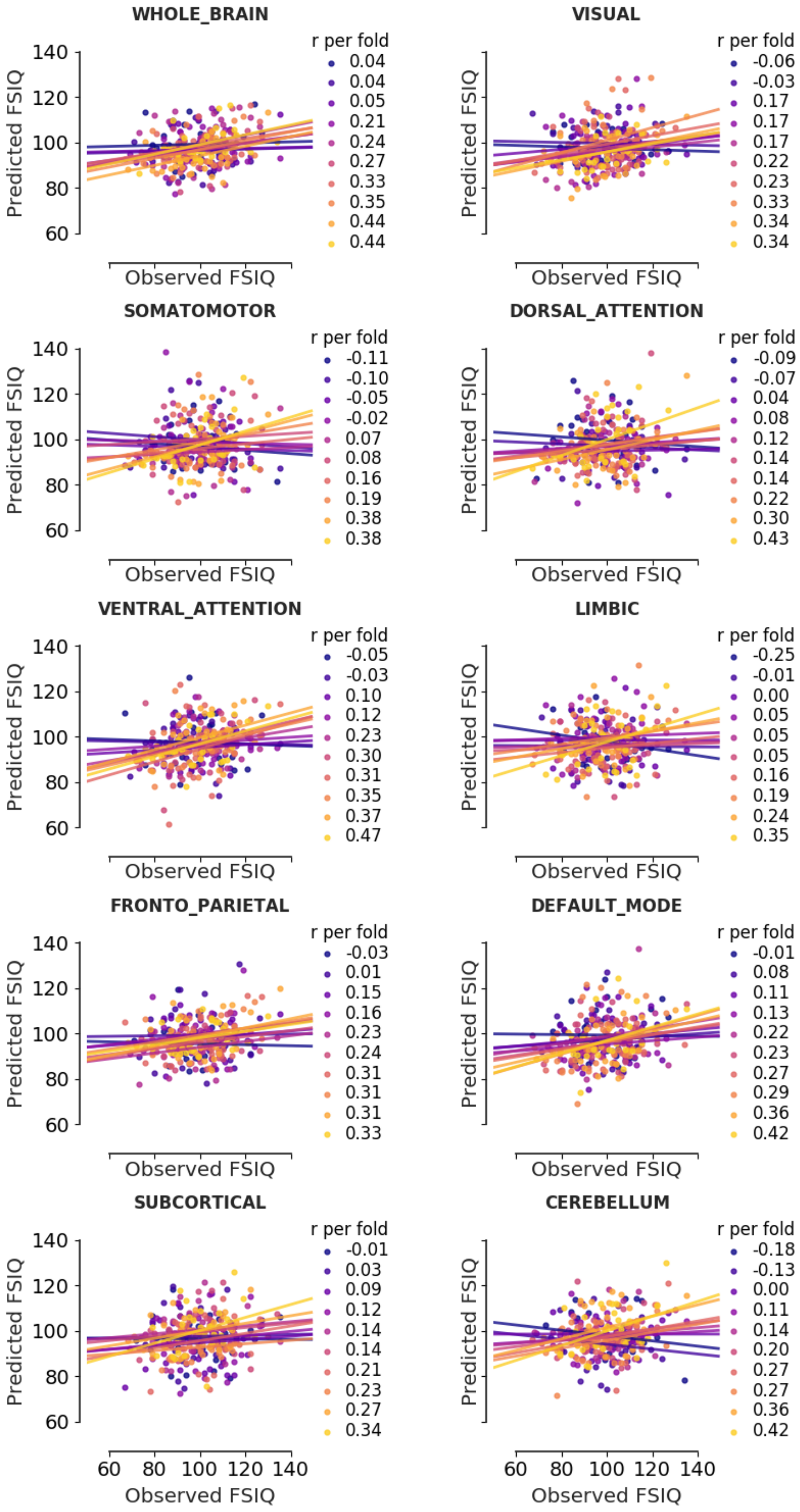

Supplementary Fig. S10 Fold-wise predictive performance for the *global* (whole brain) prediction model and all nine functional network models (*local* models) based on *absolute* gray matter volume, i.e., without correction for individual differences in brain size, and the PCA-based feature construction approach. Observed (*x*-axis) versus predicted (*y*-axis) full scale intelligence quotient (FSIQ) scores for all 308 participants based on all *absolute* gray matter volume values of the brain. Predictions of each cross-validation fold and the corresponding approximated linear regression slopes are highlighted in different colors. *r* Pearson's correlation coefficients between predicted and observed FSIQ score

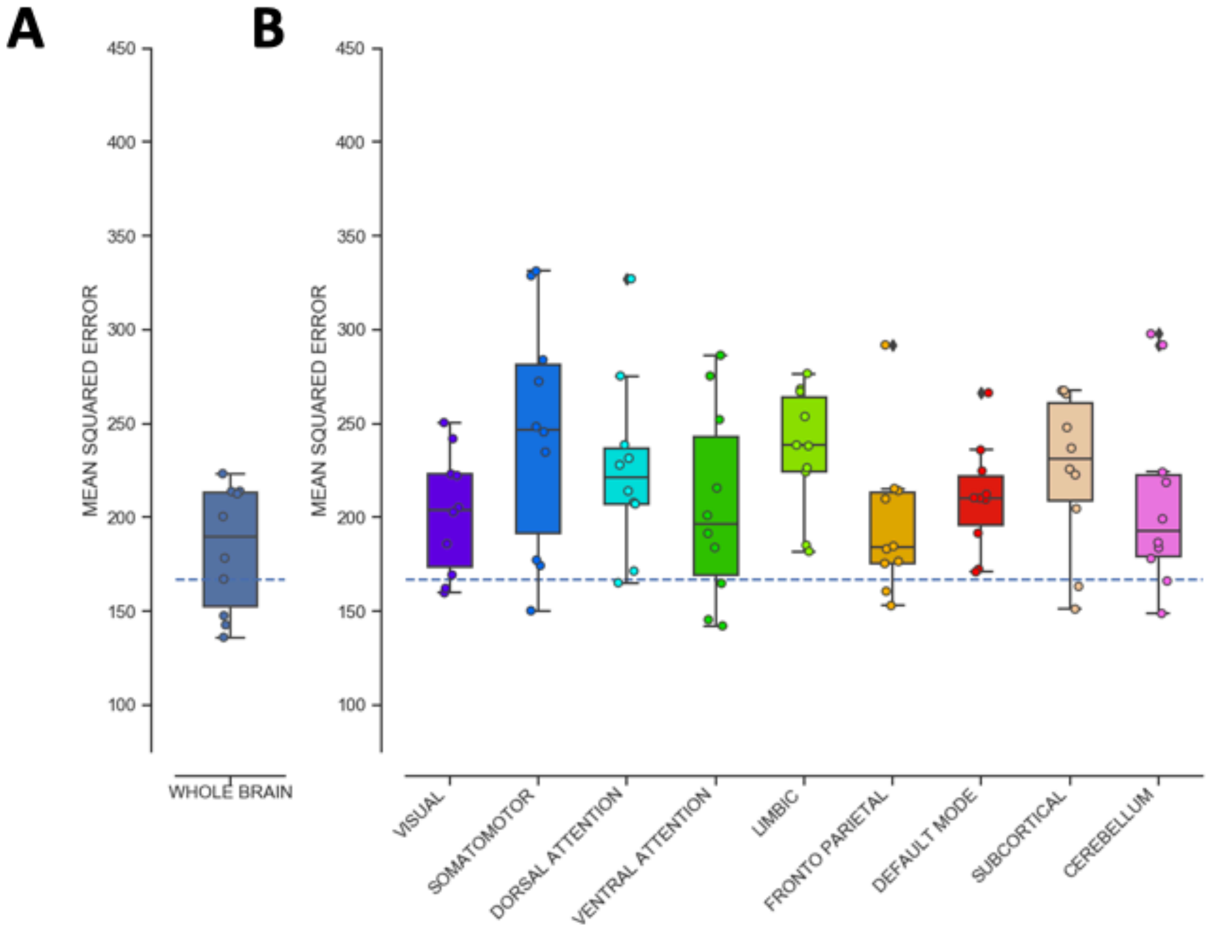

Supplementary Fig. S11 Boxplot illustrating variability of predictive performance (mean squared error, *MSE*) across cross-validation folds for *global* and *local* prediction models based on *absolute* gray matter volume, i.e., without correction for individual differences in brain size, and the PCA-based feature construction approach. The boxes represent the interquartile range, horizontal lines represent the median, and the whiskers extend to points that lie within 1.5 times the interquartile ranges. Predictive performance (*MSE*) resulted from models that were trained on data of the whole brain (**A**) and the nine functional brain networks separately (**B**). Different networks are depicted in different colors (see also Fig. 2 in the main text). The blue dotted line illustrates the performance of a ‘dummy model’ predicting the group-mean IQ of the training sample for every subject of the test sample

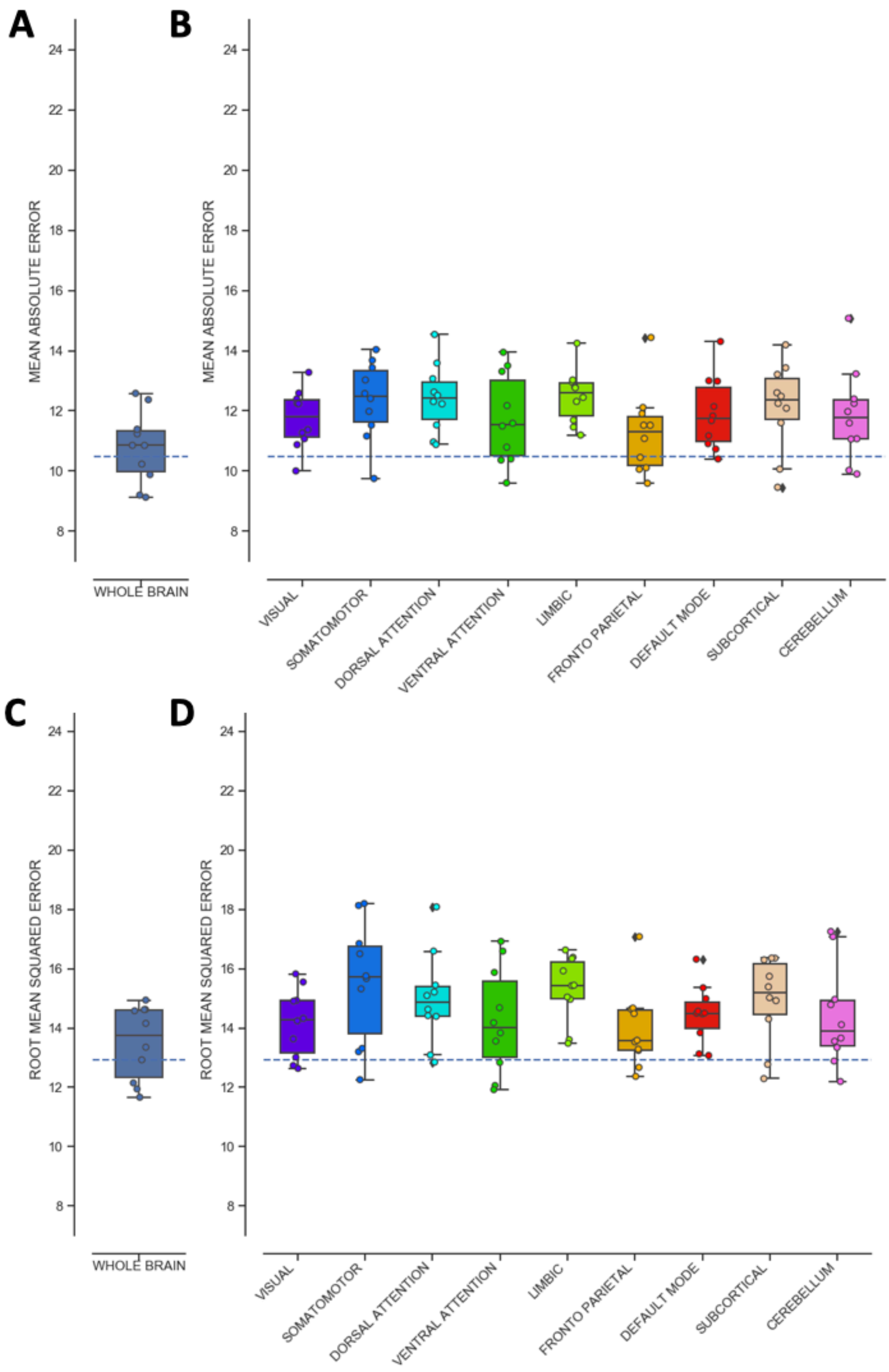

Supplementary Fig. S12 Boxplot illustrating variability of predictive performance across cross-validation folds for prediction models based on *absolute* gray matter volume, i.e., without correction for individual differences in brain size, and the PCA-based approach for the mean absolute error (*MAE*) and the root mean squared error (*RMSE*). The boxes represent the interquartile range, horizontal lines represent the median, and the whiskers extend to points that lie within 1.5 times the interquartile ranges. Predictive performance (*MAE* above, *RMSE* below) resulted from models that were trained on data of the whole brain (**A,C**) and the nine functional brain networks separately (**B,D**). Different networks are depicted in different colors (see also Fig. 2 in the main text). The blue dotted line illustrates the performance of a 'dummy model' predicting the group-mean IQ of the training sample for every subject of the test sample

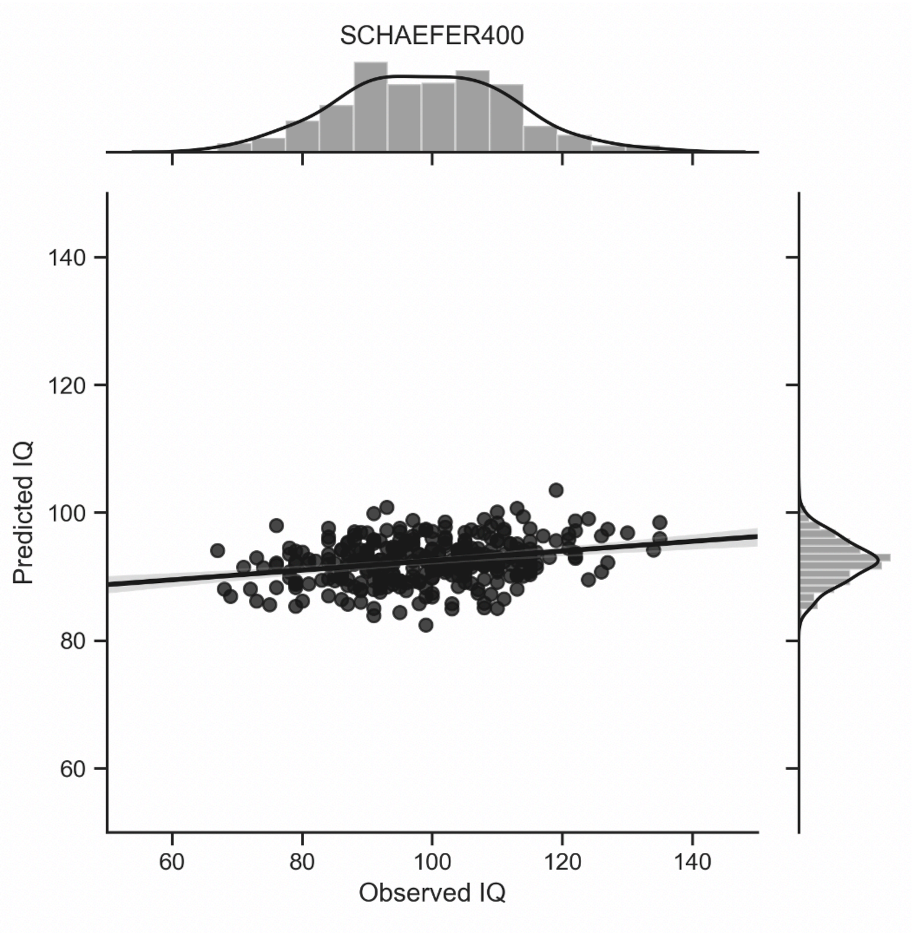

Supplementary Fig. S13 Predictive performance of the *global* model based on *absolute* gray matter volume, i.e., without correction for individual differences in brain size, and the atlas-based approach. Observed (*x*-axis) versus predicted (*y*-axis) full scale intelligence quotient (FSIQ) scores for all 308 participants. The gray area around the regression line represents the 95%-confidence interval (determined by bootstrapping) of prediction accuracy

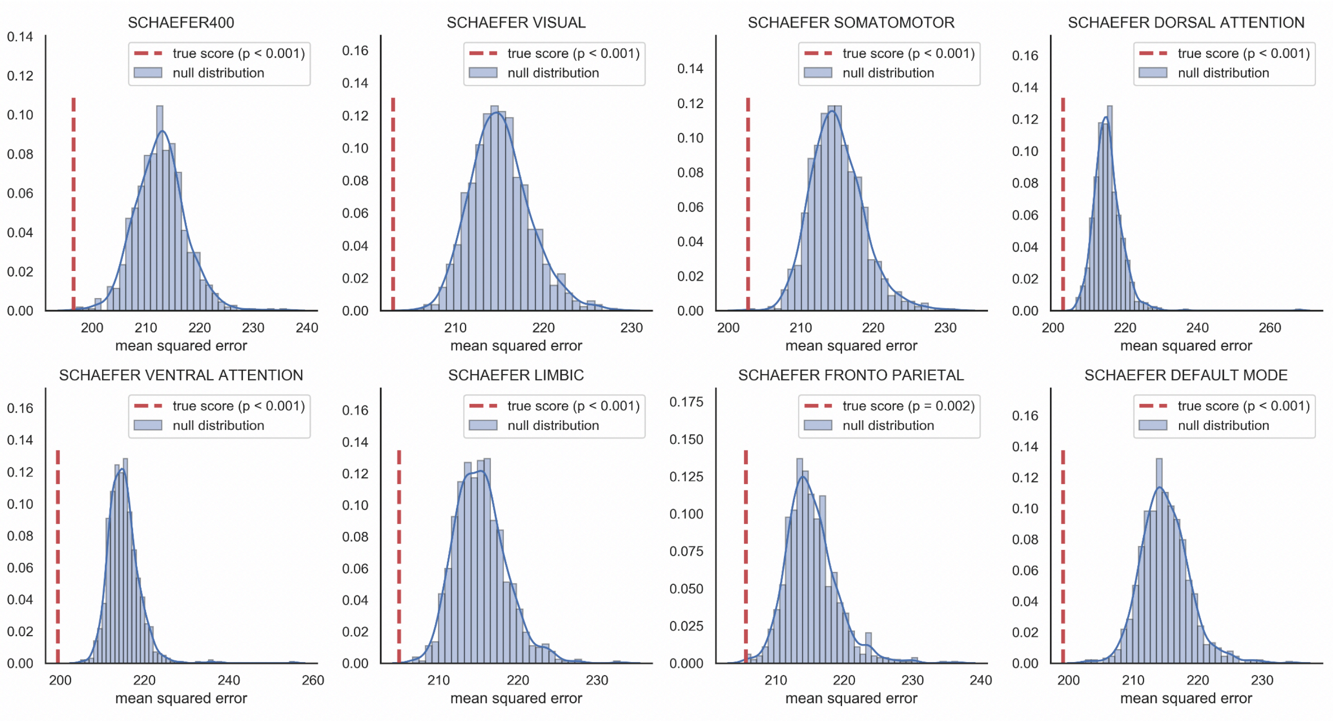

Supplementary Fig. S14 Results of the non-parametric permutation tests for the *global* (whole brain) prediction model and all seven functional network models (*local* models) based on *absolute* gray matter volume, i.e., without correction for individual differences in brain size, and the atlas-based feature construction approach. The histogram shows the predictive performance given surrogate-null data, i.e., the distribution of the test statistic (mean squared error, *MSE*) based on permuted data ($N = 1{,}000$ permutations, KDE smoothing: blue line) in relation to the predictive performance (*MSE*) based on the observed (non-permuted) data (red vertical line). If the *MSE* of the observed data had occurred in the extreme tails of the surrogate/permuted data, the prediction result from the machine learning pipeline would have been highly unlikely to be generated by chance, and thus considered significant. The *p*-values resulted from summing of the times in which model performance based on the true targets was lower than model performance based on the permuted targets and dividing this number by the number of permutations, i.e., 1,000. *p*-values correspond to the percentile position of the observed *MSE* in the distribution of surrogate-null values

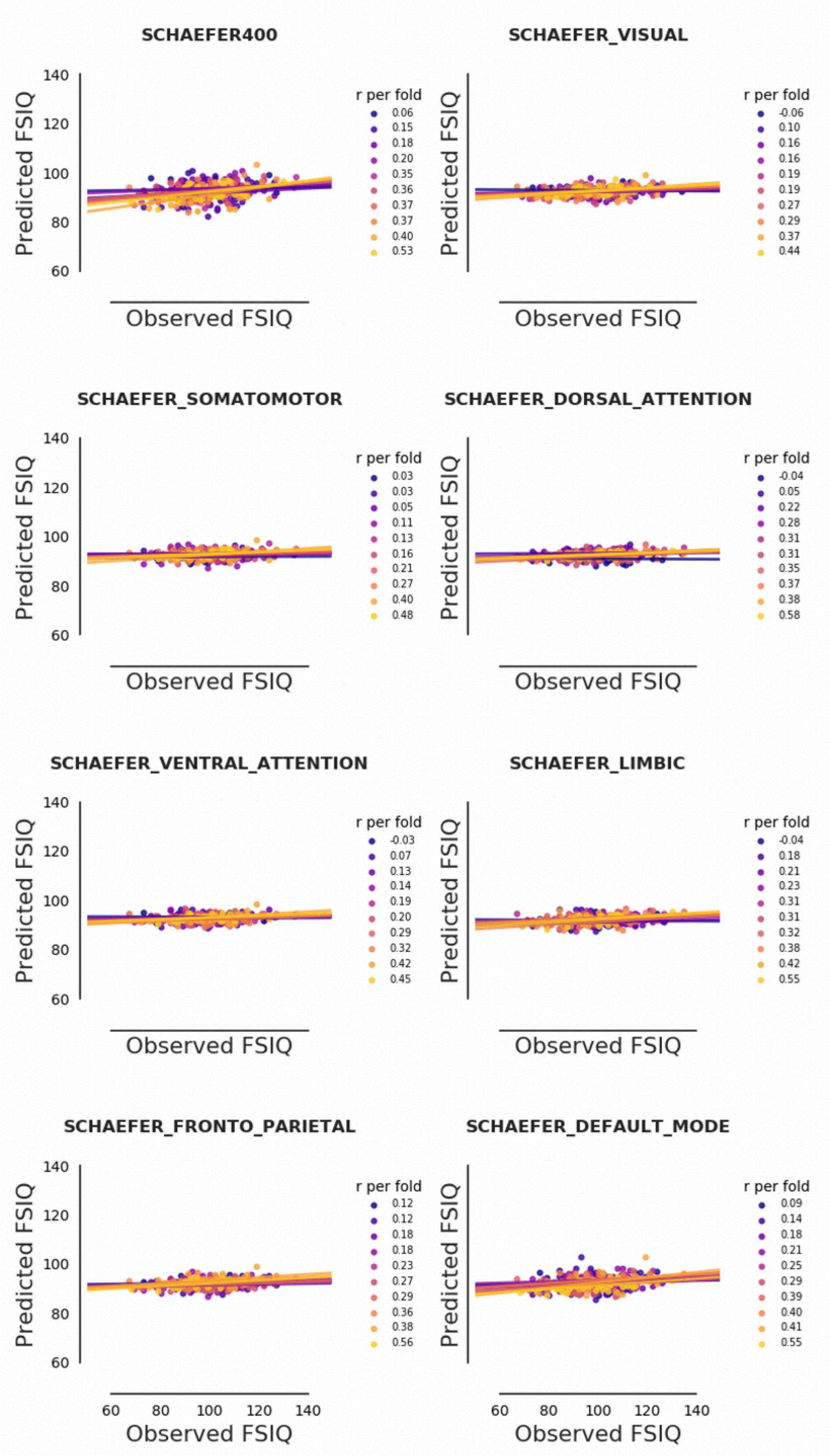

Supplementary Fig. S15 Fold-wise predictive performance based on *absolute* gray matter volume, i.e., without correction for individual differences in brain size, and the atlas-based feature construction approach. Observed (x-axis) versus predicted (y-axis) full scale intelligence quotient (FSIQ) scores for all 308 participants based on all *absolute* gray matter volume values of the brain. Predictions of each cross-validation fold and the corresponding approximated linear regression slopes are highlighted in different colors. r Pearson's correlation coefficients between predicted and observed FSIQ score

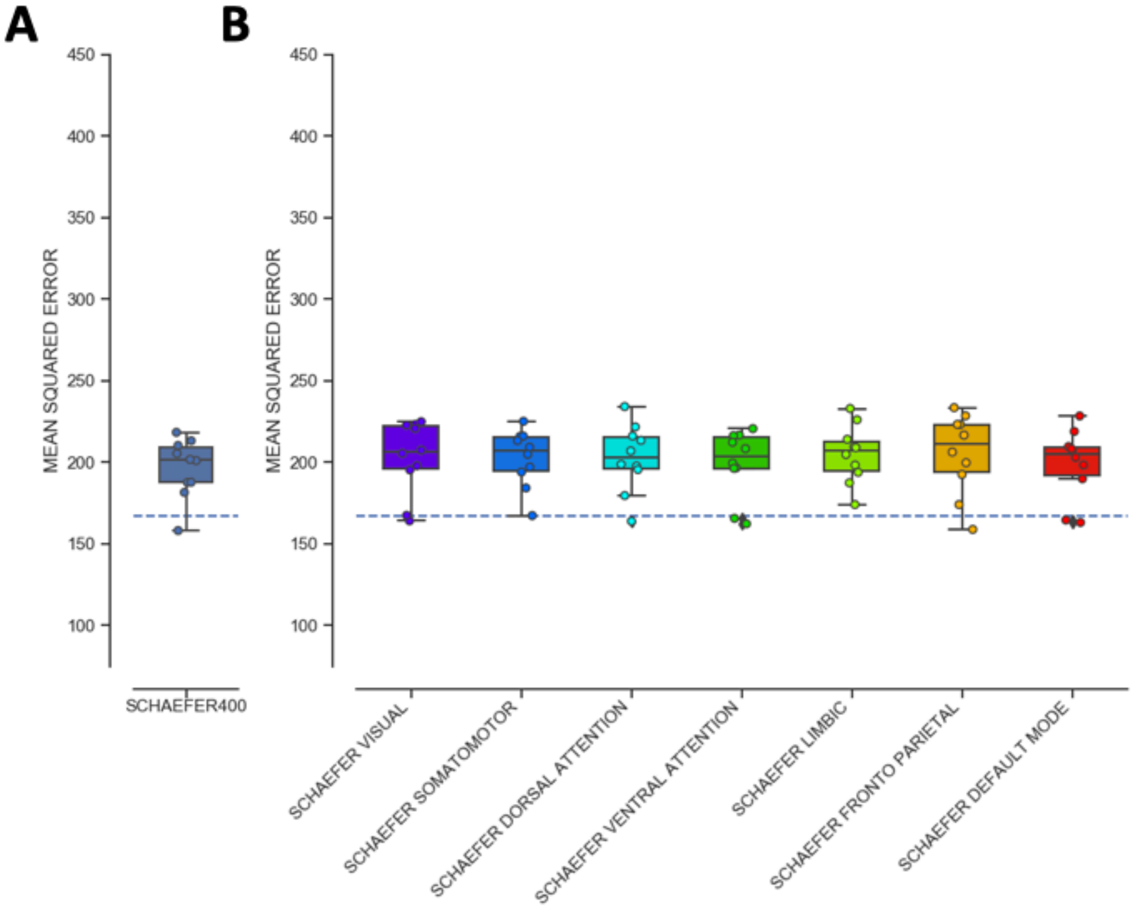

Supplementary Fig. S16 Boxplot illustrating variability of predictive performance (mean squared error, *MSE*) across cross-validation folds for prediction models based on *absolute* gray matter volume, i.e., without correction for individual differences in brain size, and atlas-based feature construction. The boxes represent the interquartile range, horizontal lines represent the median, and the whiskers extend to points that lie within 1.5 times the interquartile ranges. Predictive performance (*MSE*) resulted from models that were trained on data of the whole brain (**A**) and the nine functional brain networks separately (**B**). Different networks are depicted in different colors (see also Fig. 2 in the main text). The blue dotted line illustrates the performance of a 'dummy model' predicting the group-mean IQ of the training sample for every subject of the test sample

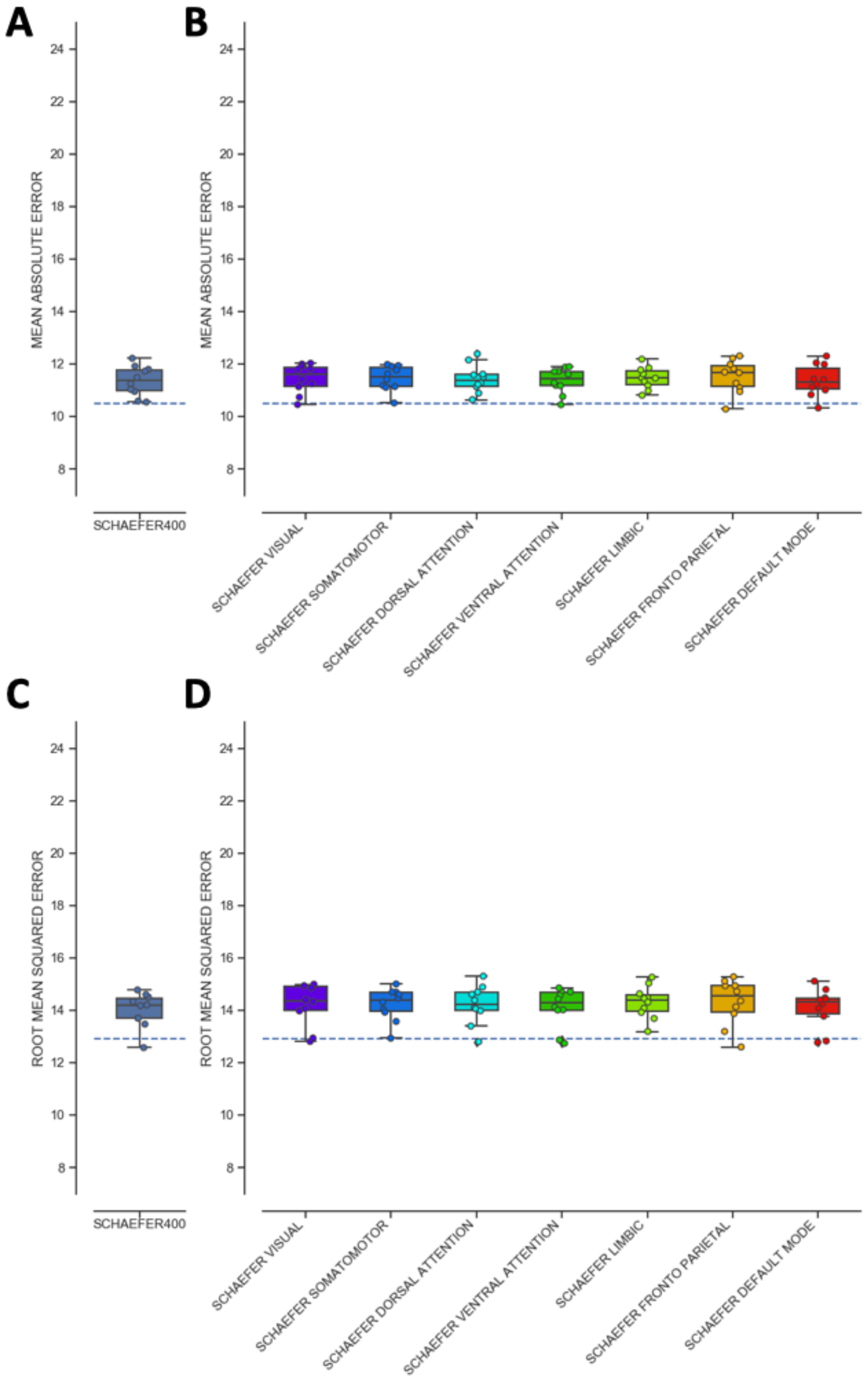

Supplementary Fig. S17 Boxplot illustrating variability of predictive performance across cross-validation folds for prediction models based on *absolute* gray matter volume, i.e., without correction for individual differences in brain size, and the atlas-based feature construction approach for the mean absolute error (*MAE*) and the root mean squared error (*RMSE*). The boxes represent the interquartile range, horizontal lines represent the median, and the whiskers extend to points that lie within 1.5 times the interquartile ranges. Predictive performance (*MAE* above*, RMSE* below) resulted from models that were trained on data of the whole brain (**A,C**) and the nine functional brain networks separately (**B,D**). Different networks are depicted in different colors (see also Fig. 2 in the main text). The blue dotted line illustrates the performance of a 'dummy model' predicting the group-mean IQ of the training sample for every subject of the test sample

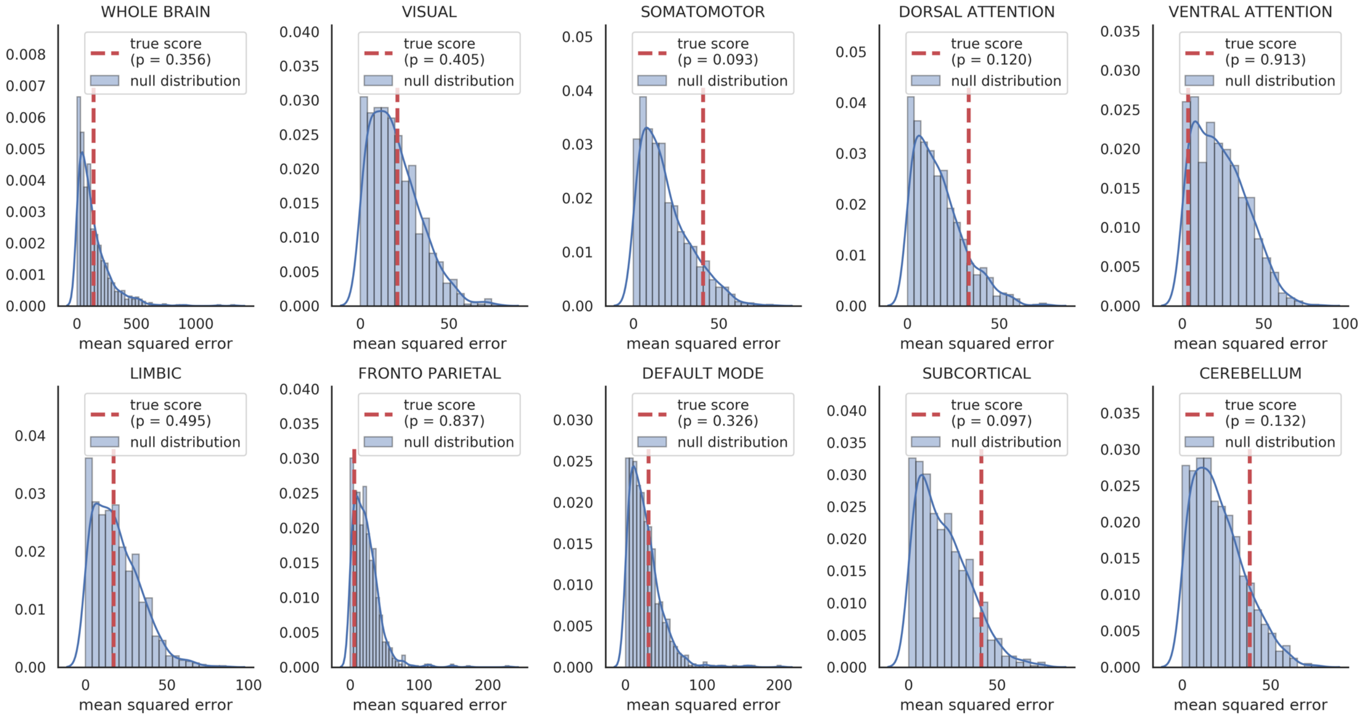

Supplementary Fig. S18 Results of the non-parametric permutation tests for model differences resulting from the PCA-based feature construction approach. Significance of difference in predictive performance between models based on *relative* gray matter volume and models based on *absolute* gray matter volume, i.e., with and without correction for individual differences in brain size. The histogram shows the predictive performance given surrogate-null data, i.e., the distribution of the test statistic (mean squared error, *MSE*) based on permuted data (*N* = 1,000 permutations, KDE smoothing: blue line) in relation to the predictive performance (*MSE*) based on the observed (non-permuted) data (red vertical line). If the *MSE* of the observed data had occurred in the extreme tails of the surrogate/permuted data, the prediction result from the machine learning pipeline would have been highly unlikely to be generated by chance, and thus considered significant. The *p*-values correspond to the percentile position of the observed *MSE* in the distribution of surrogate-null values

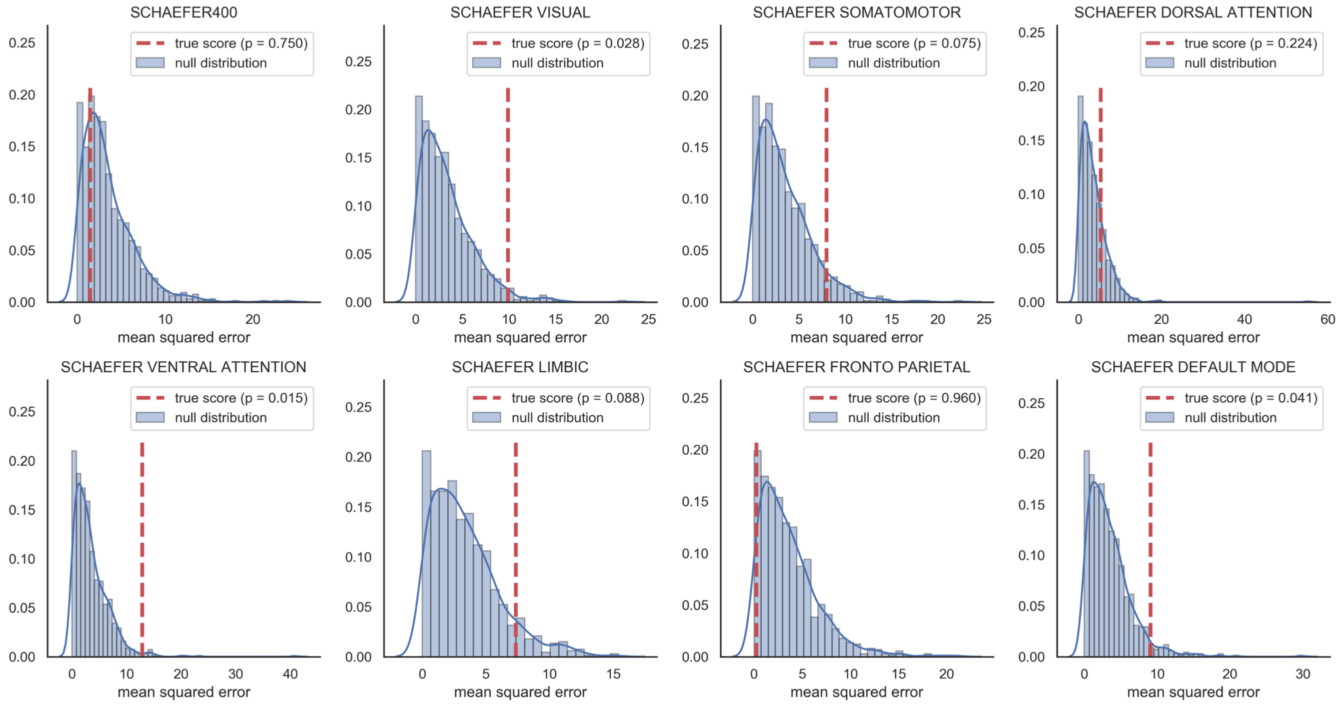

Supplementary Fig. S19 Results of the non-parametric permutation tests for model differences resulting from the atlas-based feature construction approach. Significance of difference in predictive performance between models based on *relative* gray matter volume and models based on *absolute* gray matter volume, i.e., with and without correction for individual differences in brain size. The histogram shows the predictive performance given surrogate-null data, i.e., the distribution of the test statistic (mean squared error, *MSE*) based on permuted data (*N* = 1,000 permutations, KDE smoothing: blue line) in relation to the predictive performance (*MSE*) based on the observed (non-permuted) data (red vertical line). If the *MSE* of the observed data had occurred in the extreme tails of the surrogate/permuted data, the prediction result from the machine learning pipeline would have been highly unlikely to be generated by chance, and thus considered significant. The *p*-values correspond to the percentile position of the observed *MSE* in the distribution of surrogate-null values

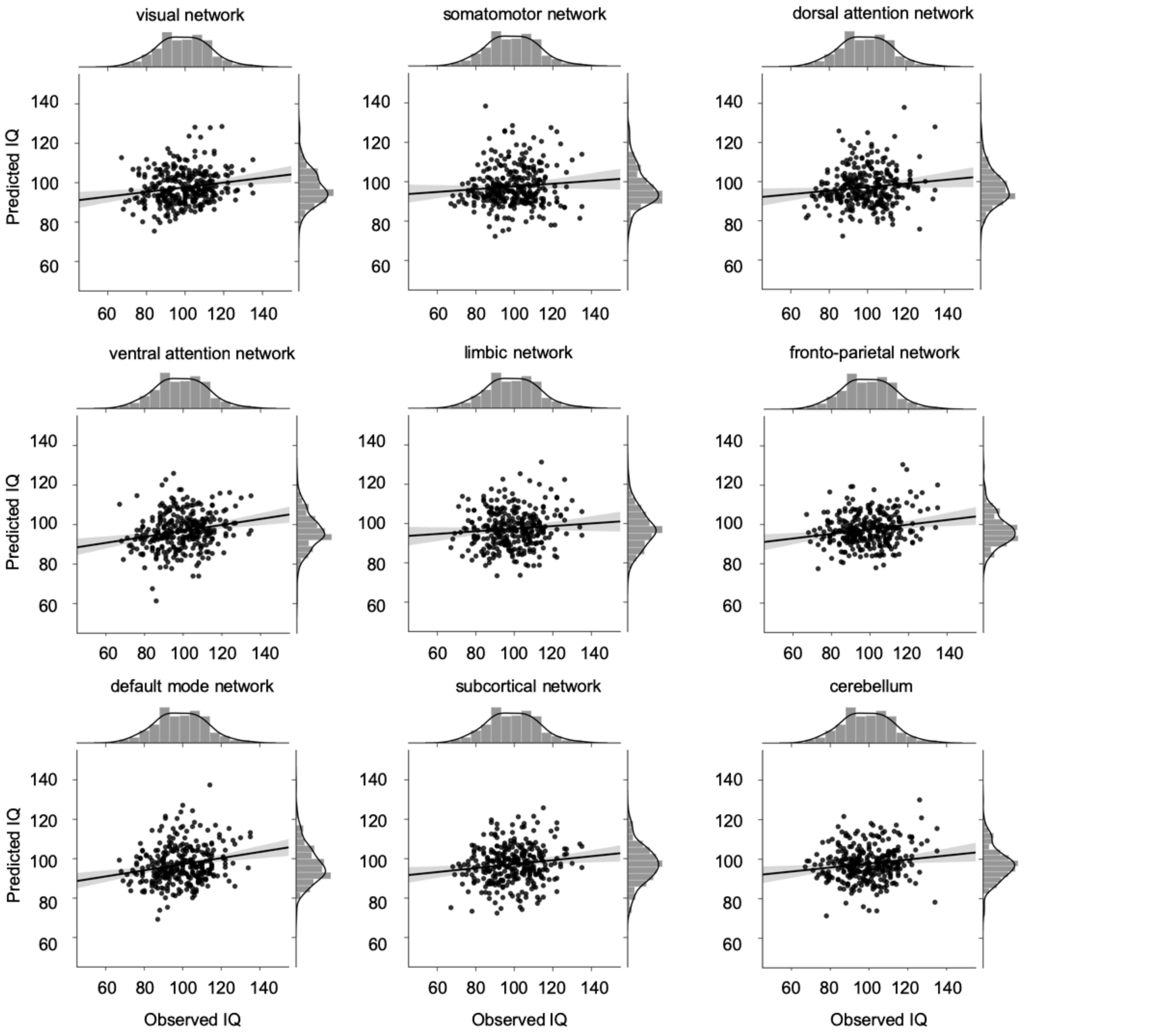

Supplementary Fig. S20 Predictive performance of the *local* model based on *absolute* gray matter volume, i.e., without correction for individual differences in brain size, and the PCA-based feature construction approach. Observed (*x*-axis) versus predicted (*y*-axis) full scale intelligence quotient (FSIQ) scores for all 308 participants. The gray areas around the regression lines represent the 95%-confidence intervals (determined by bootstrapping) of prediction accuracies

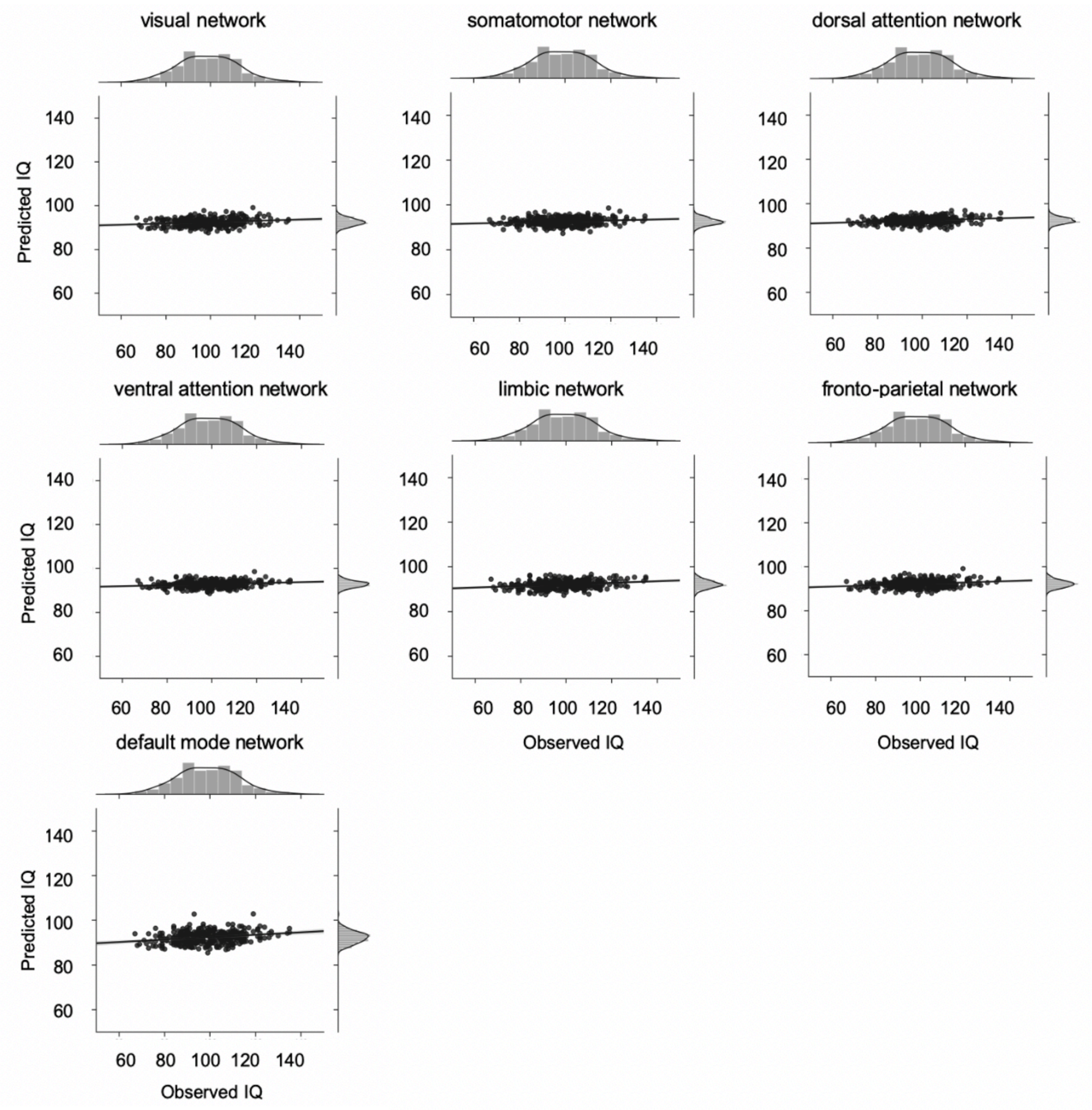

Supplementary Fig. S21 Predictive performance of the *local* model based on *absolute* gray matter volume, i.e., without correction for individual differences in brain size, and the atlas-based feature construction approach. Observed (*x*-axis) versus predicted (*y*-axis) full scale intelligence quotient (FSIQ) scores for all 308 participants. The gray areas around the regression lines represent the 95%-confidence intervals (determined by bootstrapping) of prediction accuracies

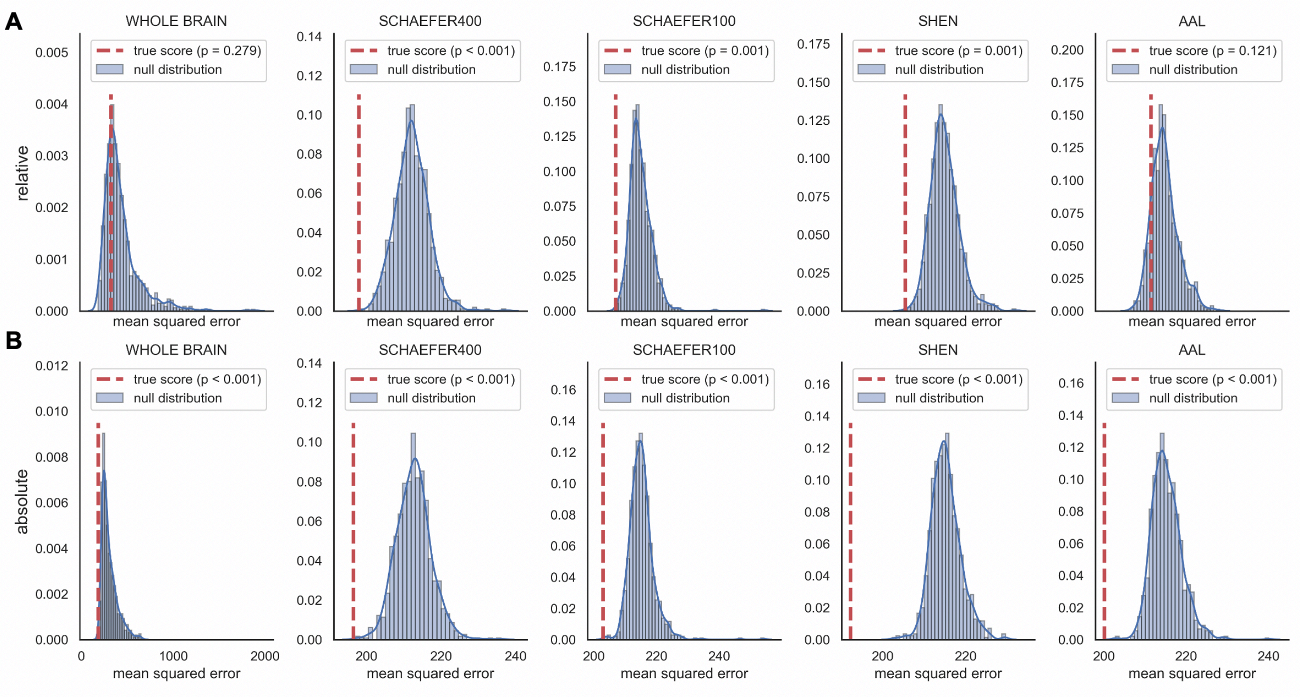

Supplementary Fig. S22 Results of the non-parametric permutation tests for *global* prediction models (control analysis using additional atlases) based on *relative* (panel A) and *absolute* (panel B) gray matter volume. The histogram shows the predictive performance given surrogate-null data, i.e., the distribution of the test statistic (mean squared error, *MSE*) based on permuted data ($N = 1{,}000$ permutations, KDE smoothing: blue line) in relation to the predictive performance (*MSE*) based on the observed (non-permuted) data (red vertical line). If the *MSE* of the observed data had occurred in the extreme tails of the surrogate/permuted data, the prediction result from the machine learning pipeline would have been highly unlikely to be generated by chance, and thus considered significant. The *p*-values resulted from summing of the times in which model performance based on the true targets was lower than model performance based on the permuted targets and dividing this number by the number of permutations, i.e., 1,000. *p*-values correspond to the percentile position of the observed *MSE* in the distribution of surrogate-null values

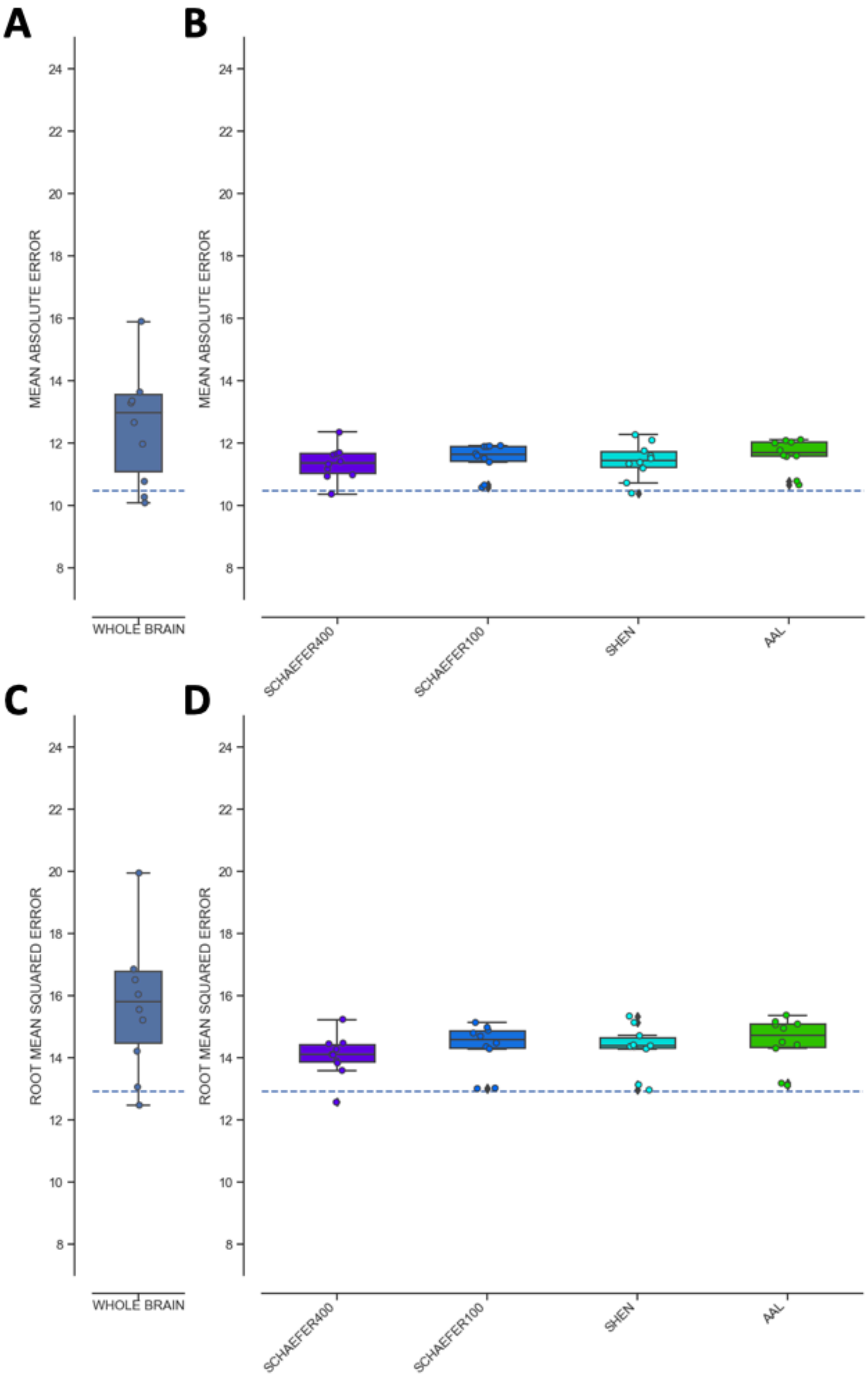

Supplementary Fig. S23 Mean absolute error (*MAE*) and root mean squared error (*RMSE*) results for prediction models based on *relative* gray matter volume (control analysis using additional atlases). Boxplots illustrating the variability of predictive performance (upper row: *MAE*; lower row: *RMSE*) across cross-validation folds for the PCA-derived *global* model (**A,C**) and the *global* models based on four additional atlases separately (**B,D**). The boxes represent the interquartile range, horizontal lines represent the median, and the whiskers extend to points that lie within 1.5 times the interquartile ranges. The blue dotted line illustrates the performance of a 'dummy model' predicting the group-mean IQ of the training sample for every subject of the test sample

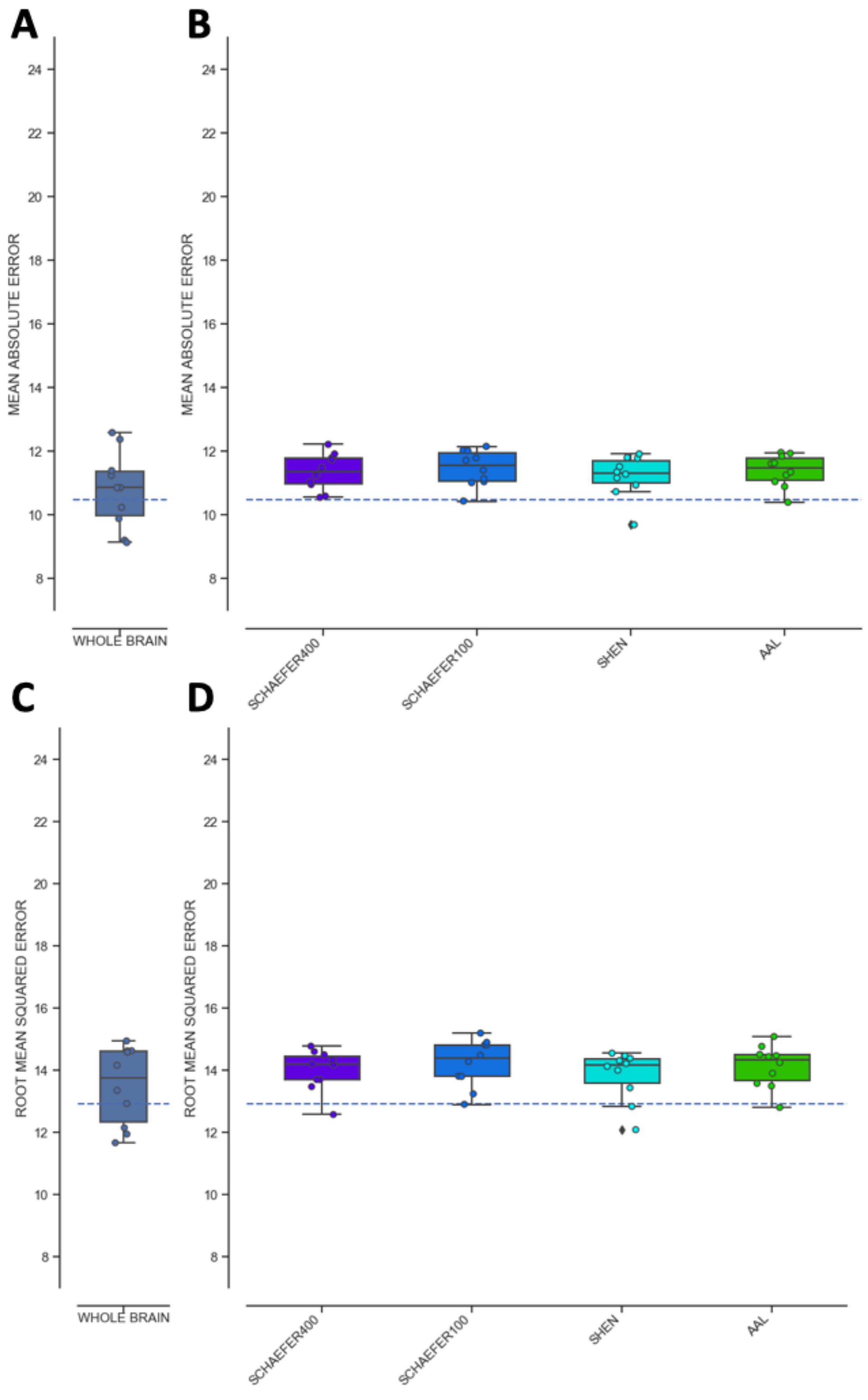

Supplementary Fig. S24 Mean absolute error (*MAE*) and root mean squared error (*RMSE*) results for prediction models based on *absolute* gray matter volume (control analysis using additional atlases). Boxplots illustrating the variability of predictive performance (upper row: *MAE*; lower row: *RMSE*) across cross-validation folds for the PCA-derived *global* model (**A,C**) and the *global* models based on four additional atlases separately (**B,D**). The boxes represent the interquartile range, horizontal lines represent the median, and the whiskers extend to points that lie within 1.5 times the interquartile ranges. The blue dotted line illustrates the performance of a 'dummy model' predicting the group-mean IQ of the training sample for every subject of the test sample
